# Supplementary material for: Spatial variation of heart failure and air pollution in Warwickshire, UK: an investigation of small scale variation at the ward-level
Source: BMJ Open. 2014 Dec 2;4(12):e006028. doi: 10.1136/bmjopen-2014-006028 (PMC4256640; doi:10.1136/bmjopen-2014-006028)
Supplement: Author's manuscript [file bmjopen-2014-006028.draft_revisions.pdf]

BMJ Open

Spatial variation of Heart Failure and Air Pollution in Warwickshire: An investigation of small scale variation at the ward-level

Journal:	*BMJ Open*
Manuscript ID:	bmjopen-2014-006028
Article Type:	Research
Date Submitted by the Author:	02-Jul-2014
Complete List of Authors:	Bennett, Oscar; Warwickshire County Council, Public Health Warwickshire,Communities Group<br>Kandala, Ngianga-Bakwin; University of Warwick, Warwick Medical School<br>Ji, Chen; University of Warwick, Warwick Medical School<br>Linnane, John; Warwickshire County Council, Public Health Warwickshire,Communities Group<br>Clarke, Aileen; University of Warwick, Division of Health Sciences
**Primary Subject Heading**:	Epidemiology
Secondary Subject Heading:	Cardiovascular medicine, Epidemiology, Public health
Keywords:	Heart failure < CARDIOLOGY, EPIDEMIOLOGY, SOCIAL MEDICINE

SCHOLARONE™
Manuscripts

For peer review only

Word count, abstract: 310

Word count, text: 3,562

References: 14

Tables: 1

Figures: 6

Spatial variation of Heart Failure and Air Pollution in Warwickshire: An investigation of small scale variation at the ward-level

Oscar Bennett MB BChir, Ngianga-Bakwin Kandala PhD[1], Chen Ji PhD[1], John Linnane MD[2], Aileen Clarke MD PhD[1].

[1] Division of Health Sciences, University of Warwick Medical School, Coventry, CV4 7AL, UK.

[2] Public Health Warwickshire, Communities Group, Warwickshire County Council ,Shire Hall, Barrack Street, Warwick, CV34 4RL, UK.

Running title: Heart Failure and Air Pollution in Warwickshire

Keywords: Warwickshire, Heart Failure, Air Pollution, epidemiology, spatial analyses

Correspondence to:

Ngianga-Bakwin Kandala, PhD
Division of Health Sciences
University of Warwick Medical School
Medical School Building
Gibbet Hill Campus
Coventry CV4 7AL
United Kingdom
Tel: + 44 (0) 2476150541
Fax:+ 44 (0) 2476528375
Email: N-B.Kandala@warwick.ac.uk

For peer review only - http://bmjopen.bmj.com/site/about/guidelines.xhtml

Abstract

Objectives: To spatially map the morbidity and mortality caused by heart failure within Warwickshire to characterise and quantify any influence of air pollution on these risks.

Design: Cross-sectional.

Setting: Warwickshire

Participants: Data from all of the 105 current Warwickshire County wards were collected on admission and death in hospital of heart failure.

Interventions: N/A

Primary and secondary outcome measures: Air pollution, heart failure hospital admissions and mortality data within Warwickshire were.

Results

In multivariate analyses, the presence of a higher NOx in a ward [3.35:1.89, 4.99], Ben [31.9:8.36, 55.85] and IMD [0.02: 0.01, 0.03], were consistently associated with a higher risk of heart failure morbidity. Pm [-12.93:-20.41, -6.54] was negatively associated with the risk of heart failure morbidity but with no association with So2.

The risk of heart failure mortality was higher in wards with a higher NOx [4.30:1.68, 7.37] and wards with more inhabitants 50+ years old [1.60: 0.47, 2.92]. Pm was negatively associated [-14.69: -23.46, -6.50] with heart failure mortality. So2, Ben, and IMD score were not associated with heart failure mortality.

There was a striking variation in heart failure morbidity and mortality risk across wards, the highest risk being in the regions around Nuneaton and Bedworth.

Conclusion

This study showed distinct spatial patterns in heart failure morbidity and mortality, suggesting the potential role of environmental factors beyond individual-level risk factors. Air pollution levels should therefore be taken into account when considering the wider determinants of public health and the impact that changes in air pollution might have on the health of a population.

For peer review only - http://bmjopen.bmj.com/site/about/guidelines.xhtml

ARTICLE FOCUS

- Air pollution has been linked to the development and exacerbation of a number of health problems including cardiovascular diseases such as heart failure.
- Heart failure is a serious condition that unfortunately affects many people in the UK. This link seems especially clear for cardiovascular and respiratory diseases such as ischaemic heart disease, heart failure, asthma, influenza, and lung cancer.
- It is well established that the ambient levels of air pollution in a region can have an impact on the health status of the population which inhabits it

KEY MESSAGES

1. Using a novel approach, this study has characterised and quantified the potential role of environmental factors (air pollutants) at the county-level.

2. The presence of a higher Mono-nitrogen Oxide (NOx) in a ward, benzene and poor Index of Multiple Deprivation (IMD) score were consistently associated with a higher risk of heart failure morbidity. Particulate matter (Pm) was negatively associated with the risk of heart failure morbidity.
3. The risk of heart failure mortality was higher in wards with a higher NOx and wards with more inhabitants 50+ years old. Pm was negatively associated with heart failure mortality.
4. There was a striking variation in heart failure morbidity and mortality risk across wards, the highest risk being in the regions around Nuneaton and Bedworth.

STRENGTHS AND LIMITATIONS

- This study showed distinct spatial patterns in heart failure morbidity and mortality in Warwickshire, suggesting the potential role of environmental factors beyond individual-level risk factors.
- Air pollution levels should therefore be taken into account when considering the wider determinants of public health and the impact that changes in air pollution might have on the health of a population

For peer review only

For peer review only - http://bmjopen.bmj.com/site/about/guidelines.xhtml

Introduction

Air pollution has been linked to the development and exacerbation of a number of health problems. This link seems especially clear for cardiovascular and respiratory diseases such as ischaemic heart disease, heart failure, asthma, influenza, and lung cancer [1, 2, 3, 4]. It is well established that the ambient levels of air pollution in a region can have an impact on the health status of the population which inhabits it [5]. Air pollution levels should therefore be taken into account when considering the wider determinants of public health and the impact that changes in air pollution might have on the health of a population.

The issue of air pollution has been highlighted in Warwickshire recently by the setting up of Air Quality Management Areas (AQMAs) in a number of different parts of the county. These are specific areas in the county that have been identified as places where, without a focused local council strategy to reduce air pollution, future government targets for air pollutant concentrations may not be met. Within Warwickshire the specific air pollutant that has been identified as a problem and led to these special areas being setup is mono-nitrogen oxides (NOx)[6].

The objective of the present study was therefore to look for any relationship between levels of air pollution and the morbidity and mortality associated with heart failure within Warwickshire in the last few years (2005-2013 for morbidity, 2007-2012 for mortality). We examined a range of traditional air pollutants for an ecological association with heart failure morbidity and mortality at the county level. Furthermore, the present analysis attempted to highlight spatial patterns in heart failure morbidity and mortality risk within the county, after multiple adjustments for proximate, county-level factors. The geographic locations of wards within the county can be considered as proxy measures of many other unmeasured factors such as availability and access to health services, individual health seeking behaviour, preventive ward policy and general ward factors. Such estimates might illustrate how much

For peer review only - http://bmjopen.bmj.com/site/about/guidelines.xhtml

can be learned by detailed exploratory analyses as well as how these data can be used to strategically inform policy aiming at the prevention and management of serious conditions such as heart failure in these settings.

The evidence from published literature for the link between air pollution and health problems

Studies have shown an effect on the health of populations caused by both long term exposure as well as short term "spikes" in local air pollution levels[5].

A relevant systematic review and meta-analysis has been published recently in the Lancet [2]. It pooled the results of studies that have been conducted worldwide looking at the temporal relationship between the levels of a number of different air pollutants with local heart failure hospital admission rates and mortality rates. This showed a clear link between short term rises in all types of air pollution (except ozone) and rises in hospital admissions and mortality due to heart failure. This study did not look at any potential effects from long term exposure to air pollution. However it did provide strong evidence that air pollution can 'exacerbate' heart failure, increasing the likelihood that a patient with existing heart failure will become sufficiently unwell to require hospital admission, or even die.

The ESCAPE study[3], published recently in the Lancet Oncology, pooling the results of 17 cohort studies from around Europe looked at the ambient levels of air pollution in an area (particulate matter and NOx) and the incidence of lung cancers in the inhabitants of this area during many years of follow up (mean 12.8 years). This was intended to look at the risk associated with long term exposure to air pollution. A statistically significant correlation was found between the levels of particulate matter air pollution and the local incidence of lung cancer diagnoses. Particulate matter with a diameter of less than 10 µm had a hazard ratio of 1.22 [95% CI 1.03-1.45] per (10 µg/cubic meter). In other words, for every increase in particulate matter pollution of 10 µg/cubic meter there was a corresponding immediate

For peer review only - http://bmjopen.bmj.com/site/about/guidelines.xhtml

increase in the chance of being diagnosed with lung cancer of 22% (95% CI 3% - 45%). There was also a correlation between traffic volume within 100m of a residence and a modest increase in the rate of lung cancer (Hazard ratio 1.09 (CI 0.99-1.21)). No similar correlation was found with NOx air pollution. This suggests that long term exposure to higher levels of particulate matter air pollution may increase the incidence of lung cancer in a population. Another study published in the United States in 2004 looked at the long term effect of exposure to particulate matter air pollution and the mortality attributed to different cardiovascular and respiratory diseases in different areas of the United States[(4)]. A good correlation was found between the degree of long term exposure to particulate matter air pollution and increases in mortality from cardiovascular diseases, including heart failure. Interestingly this was not found to be the case for most respiratory diseases. There was also an element of the study that looked at the effect of a person's smoking status on the mortality statistics. This found, as expected, a strong link between mortality and smoking. However it also found that air pollution contributed an additional rise in cardiovascular mortality on top of that attributed to smoking which was at least additive if not synergistic as a risk factor.

Methods

Study data

In order to carry out this project data was collected about the geographical distribution of air pollution within Warwickshire. This data included information about each of four individual components of air pollution (NOx, sulphur dioxide, particulate matter, and benzene) which could then be united into a combined index (all of the contributions added together). A single recorded level from 2010 of each air pollutant for each ward was used in the study. This was then compared to collected data about:

(i) The geographical distribution of home addresses of patients who were admitted to hospital because of heart failure or a complication of heart failure. Hospital

For peer review only - http://bmjopen.bmj.com/site/about/guidelines.xhtml

admission rate in an area due to heart failure was used as a proxy indicator for the level of heart failure morbidity within that area.

(ii) The geographical distribution of home addresses of patients who died from heart failure, or whose death was contributed to by heart failure.

These data were collected by the Warwickshire Observatory, which is part of the Warwickshire County Council in charge of collecting and handling statistics relating to the county. Ward level population data were obtained from the 2011 census. Data from all of the 105 current Warwickshire County wards were collected.

The potential confounding variables of firstly age structure of a population within a ward and secondly levels of social deprivation within a ward were identified [7,8]. These were then controlled for in the second stage of the statistical analysis (see below).

Information about the age structure of wards was obtained from the Office of National Statistics mid 2010 estimates (obtained from the Warwickshire Observatory website)[9]. The representative statistical value of "percentage (%) of population above age 50" was used as an indicator of wards with a higher proportion of older people.

Information about social deprivation was obtained from the English Indices of Deprivation published by the Department for Communities and Local Government [10]. The Index of Multiple Deprivation (IMD) averaged across each ward was used as an indicator of the level of deprivation within the wards of Warwickshire.

The information on crude observed air pollution level distribution, heart failure hospital admission rates and mortality rates were then represented on maps of Warwickshire (**Figure 1**).

Statistical Analysis

To account for spatial autocorrelation in observed heart failure hospital admission and mortality rates at the ward level, in Warwickshire, we applied a unified approach to account

For peer review only - http://bmjopen.bmj.com/site/about/guidelines.xhtml

for possible air pollution effects of environmental risk factors. This was achieved using a geo-additive semi-parametric mixed model. The model employed a fully Bayesian approach using Markov Chain Monte Carlo (MCMC) techniques for inference and model checking [11, 12]. Response variables were defined as the count (per 1000 population) of heart failure morbidity or mortality in a ward (Poisson model): $y_i / \eta_i, \delta \sim B(\eta_i, \delta)$ for a binomial formulation. The standard measure of effect was the posterior mean (PM) and 95% credible region (CR).

The analysis was carried out using version 2.0.1 of the BayesX software package, which permits Bayesian inference based on MCMC simulation techniques[13]. Multivariate Bayesian geo-additive regression models were used to evaluate the significance of the posterior mean (PM) determined for the fixed effects and spatial effects between air pollution and the morbidity and mortality from heart failure within Warwickshire. Each component of air pollution was looked at separately and then also the combined index in unadjusted models. Next, fully adjusted multivariate Bayesian geo-additive regressions analysis were performed to look again for statistically significant correlation between these variables, but this time further controlling for any influence from age structure or social deprivation.

Results

The following maps display the observed data collected for this study using graduated colouring to represent data value categories within each ward.

Observed Air Quality Map

This map (**Figure 1left**) was produced using 2010 data from the Warwickshire Observatory. It is based on a combined air quality indicator which is a combination of information about the contribution to air pollution from NOx, sulphur dioxide, particulate matter, and benzene. The Warwickshire Observatory description of this index reads as follows:

For peer review only - http://bmjopen.bmj.com/site/about/guidelines.xhtml

"Combined Air Quality Indicator (estimates of emissions for four pollutants: benzene, nitrogen dioxide, sulphur dioxide and particulates) for small areas (modelled to 1 km grid squares) where an index value of 1 is equivalent to the national standard for each pollutant. The values are then summed so an overall score of 4 would represent all four pollutants being present at the national standard level."[(14)] These specific standards are described in the Air Quality Standards Regulations 2010.

The geographical pattern of the observed air pollution across the county shows a higher level of air pollution near the more urban centres of Birmingham and Coventry. The proximity of parts of the county to motorways such as the M6 and M42 could also be a contributing factor to the observed pattern. There was no place in the county where the level of any pollutant exceeded the national standard in 2010.

Heart Failure Hospital Admission Map

This map (**Figure 1centre**) shows the geographical distribution of the home addresses of patients admitted to hospital with a diagnosis of heart failure (or exacerbation of heart failure) within the April 2005- April 2013 period.

Heart Failure Mortality Map

This map (**Figure 1right**) shows the geographical distribution of the home addresses of patients who died either directly or in part from heart failure in the 2007-2012 (inclusive) periods.

Table 1 (left panel) displays posterior means of heart failure admission across the selected covariates following multivariate Bayesian geo-additive regression analyses.

The Posterior mean (PM) & 95% Credible Region (CR) of overall hospital admission rates due to heart failure and mortality rates from heart failure were [6.19 (3.84, 8.97)] and [4.13

For peer review only

(1.18, 7.43)] (Table 2) respectively. On average, the presence of a higher Mono-nitrogen oxide indicator (NOx) in a ward [Posterior mean (PM) & 95% Credible Region (CR): 3.35 (1.89, 4.99)], Benzene indicator (Ben) [31.9 (8.36, 55.85)] and IMD 2010 score [0.02 (0.01, 0.03)], were consistently associated with higher risk of heart failure morbidity. The particulates indicator (Pm) [-12.93 (-20.41, -6.54)] was negatively associated with the risk of heart failure morbidity and Sulphur dioxide indicator (So2) was not associated with heart failure admission.

Table 1(right panel) shows the same corresponding results for heart failure mortality. This table shows that the risk of heart failure mortality was higher in wards with a higher Mono-nitrogen oxide indicator (NOx) [4.30 (1.68, 7.37)] and wards with more inhabitants over 50 years old [1.60 (0.47, 2.92)]. The particulates indicator (Pm) was negatively associated with heart failure mortality. The sulphur dioxide indicator (So2), Benzene indicator (Ben), and IMD score were not associated with heart failure mortality.

The combined air pollution index (all indicators averaged together) when incorporated into a separate model combining age and social deprivation was significantly positively associated with both heart failure morbidity [1.39 (0.87, 1.81)] and mortality [1.79 (0.85, 2.55)] across the county.

In **Figures 2 and 4**, the left-hand maps show the unadjusted estimates air pollution posterior total residual ward means of heart failure morbidity and mortality. **Figures 3 and 5** show the adjusted PM after multiple adjustment for the geographical location, taking into account the auto-correlation structure in the data, the uncertainty in the ward level and all ward-level risk factors for heart failure morbidity and mortality. The red colour indicates the maximum posterior mean recorded while green denotes a lower mean. The right-hand maps show the 95% posterior probability of heart failure and mortality, which indicate the statistical significance associated with the total excess risk. Black colour indicates a negative spatial

For peer review only - http://bmjopen.bmj.com/site/about/guidelines.xhtml

effect (associated with increased risk of heart failure and mortality), white colour a positive effect (an decreased risk) and grey colour a non-significant effect.

In general, there was consistently higher heart failure morbidity risk in northern wards particularly around Nuneaton and Bedworth and lower heart failure morbidity risks in the southern wards particularly within the district of Stratford-on-Avon. However, all this variation could not be accounted for within the model generated within this study (taking into account air pollution, age, and social deprivation levels). On the other hand, for heart failure mortality risks, in general, heart failure mortality risk was higher in northern wards around Nuneaton and Bedworth as well as in some central areas around Warwick, Royal Leamington Spa, and Kenilworth. Heart failure mortality risk was again lower in more southern wards particularly within Stratford-on-Avon. Unlike with morbidity however, our model could not explain all this variation in heart failure mortality. Even after taking into account air pollution, age, and social deprivation the rate of death from heart failure remained significantly higher than would be expected in areas within and around Nuneaton, Bedworth, Warwick, Royal Leamington Spa, and Kenilworth.

In sensitivity analyses, we tested several models and our results were not substantially altered after removing one or two pollutant (data not shown).

Discussion

Before even considering air pollution, this study helps to demonstrate the inequality in risk from heart failure disease and death that exists for individuals living in different parts of the county of Warwickshire. There is a significant excess risk of both disease and death in more northern wards within and surrounding Nuneaton and Bedworth. Much (but not all) of this risk could be attributed to the air pollution and social deprivation that exists in these areas according to our model. Measures that seek to address air pollution and social deprivation

For peer review only - http://bmjopen.bmj.com/site/about/guidelines.xhtml

could be expected therefore to help mitigate against cardiovascular risks within these local populations.

The present study corroborates the notion that air pollution is an increasingly important public health issue in Warwickshire. Higher levels of the average air pollution index looked at in this study correlated significantly with increased levels of both heart failure morbidity and mortality, even after removing the effects of age structure and social deprivation. The individual component of NOx air pollution seems particularly to contribute risk to both the morbidity and mortality of heart failure within the county, suggesting that it may have a particularly detrimental effect on heart failure patients. This reinforces the importance of the AQMAs set up within the county in response to high NOx levels. Road traffic is a large contributor to air pollution within Warwickshire. Diesel engines are responsible for a large part of the NOx component of these emissions.

An unexpected result also appeared within our analysis. Particulate matter was positively non-significantly correlated with heart failure morbidity and mortality on its own. However it became negatively significantly correlated when incorporated into the whole model with all the other factors taken into account and controlled for in this study. This would imply some sort of unexpected "protective influence" from particulate matter air pollution on heart failure patients. This clearly contradicts our expectations and is at odds with a wealth of existing evidence that indicates that particulate matter air pollution contributes risk to and exacerbates cardiovascular disease[(4)].

A possible explanation for this is based on the following four observations:

1. The above-mentioned negative correlation of particulate matter air pollution with heart failure morbidity and mortality in our model.
2. Particulate matter air pollution actually varied very little across the county compared to the other types of air pollution. All types of air pollution tended to decrease in rural

For peer review only - http://bmjopen.bmj.com/site/about/guidelines.xhtml

areas, but particulate matter tended to decrease much *less* compared to the other components of air pollution. Consequently in rural areas of the county where most types of air pollution are significantly lower, particulate matter pollution was relatively higher compared to NOx, Benzene, and SO2.

3. There seems to be high risk of heart failure deaths in urban centres (particularly Nuneaton, Bedworth, Warwick, Royal Leamington Spa, and Kenilworth), higher than can be explained by our model.
4. Conversely, there seems to be a particularly low risk of heart failure deaths in some rural areas within the western part of Stratford-on-Avon, lower than can be explained by our model.

Our hypothesis is that there is an additional factor influencing the morbidity and mortality of heart failure not looked at in this study, namely the urban/rural nature of a patient's living environment. It could be the case that living in an urban environment contributed risk and living in a rural area provided protection against heart failure morbidity and mortality. This would be an effect in *addition* to any increase in air pollution or social deprivation within urban settings compared to rural settings. This could certainly in principle be plausible, with people in rural areas perhaps doing more physical activity, eating more healthily etc. If this were the case it would explain the excess deaths in urban centres found in this study. It could also be responsible for the unexpected protective factor attributed to particulate matter air pollution in our analysis. Given that the particulate matter component of air pollution is *relatively* higher than the other components in rural areas the protection that living in rural areas affords individuals could be misleadingly attributed to the particulate matter component of air pollution present in these areas. Further work will need to be done looking into this possible link between urban/rural living environments and heart failure morbidity and mortality. It could be very revealing to carefully characterise this effect if it indeed exists, as it

For peer review only - http://bmjopen.bmj.com/site/about/guidelines.xhtml

may be an indication of unrecognised cardiovascular risk/protective factors associated with urban/rural living that exists within Warwickshire.

There are some limitations in this study worth considering that result from assumptions made along the way. A single air pollution measurement in 2010 was used and it was assumed that there was no significant change in this value over the 2005-2013 period that mortality and hospital admission data was gathered from. The resulting cross-sectional nature of the study does not allow establishing temporality and thus causality of the observed associations. There was also no way to determine the length of time that individual members of the population within a ward had lived in that area, and thus how long they had been exposed to the measured ambient air pollution level. It was assumed that people with home addresses in a ward were exposed significantly to the levels of air pollution in that ward. It is worth noting that the correlation between NOx pollution and cardiovascular risk picked up in this piece of work is in line with the conclusions drawn by a significant number of other well powered studies using large amounts of data from all around the world[(2)].

In summary, this study has provided a number of interesting results. Firstly it has helped to quantify the inequality that exists across different parts of Warwickshire with regards to heart failure risk. It has also provided some interesting circumstantial evidence of a link between heart failure morbidity and air pollution (particularly NOx). Finally it has also given a suggestion of a possible link between living in urban environments and a higher risk of cardiovascular disease, and a corresponding lower risk from living in rural environments. More work will need to be done to look into this possibility. It would be informative to run this analysis whilst factoring in the influence of a person's distance from their nearest urban centre. This urban/rural factor should be further explored and mined for additional information as it could be an indication of hitherto unconsidered factors influencing the health

For peer review only - http://bmjopen.bmj.com/site/about/guidelines.xhtml

status of the population of Warwickshire and possibly further afield. It would also be interesting to look at the effects of air pollution variation in the shorter term. For example, looking at how local "spikes" in air pollution affect the rates of hospital admissions locally immediately following it. This could be done in Leamington Spa where there is an air quality monitoring station constantly measuring the levels of air pollutants.

Other health problems, such as ischaemic heart disease and respiratory diseases, have been linked with air pollution as well and it could be informative to also look into these links locally.

The Warwickshire maps displaying the distribution of air pollution, heart failure hospital admissions, and heart failure mortality (Figure1, 2, 3,4 respectively) provide a convenient way to see the specific areas where these problems are relatively more or less severe.

Contributorship statement:

O B Conceived the idea analyzed the data, contributed to formulating the results and wrote the first draft. N-B K analyzed the data, advised on statistical aspects, contributed to formulating the results wrote the second draft. CJ analyzed the data. J L helped coordinate the project and co-wrote the final draft; AC coordinated the project, advised on all aspects and co-wrote the final draft.

Funding:

This paper presents independent research supported by the National Institute for Health Research (NIHR) Collaborations for Leadership in Applied Health Research and Care West Midlands. The views expressed are those of the author(s) and not necessarily those of the NHS, the NIHR or the Department of Health.

Competing interests: The authors declared no conflict of interest.

Data sharing: Not application

For peer review only - http://bmjopen.bmj.com/site/about/guidelines.xhtml

References

1. Particulate Air Pollution and Hospital Emergency Room Visits for Asthma in Seattle, Joel Schwartz, Daniel Slater, Timothy V. Larson, William E. Pierson, and Jane Q. Koenig. American Review of Respiratory Disease, Vol. 147, No. 4 (1993), pp. 826-831.
2. Global association of air pollution and heart failure: a systematic review and meta-analysis, Anoop SV Shah, Jeremy P Langrish,Harish Nair,David A McAllister, Amanda L Hunter, Prof Ken Donaldson, Prof David E Newby, Dr Nicholas L Mills. The Lancet, Early Online Publication, 10 July 2013
DOI: 10.1016/S0140-6736(13)60898-3
3. Air pollution and lung cancer incidence in 17 European cohorts: prospective analyses from the European Study of Cohorts for Air Pollution Effects (ESCAPE), Raaschou-Nielsen O, Andersen ZJ, et al. Lancet Oncol. 2013 Aug;14(9):813-22
4. Cardiovascular Mortality and Long-Term Exposure to Particulate Air Pollution, Epidemiological Evidence of General Pathophysiological Pathways of Disease
C. Arden Pope III, Richard T. Burnett, et al. Circulation. 2004; 109: 71-77
5. Air pollution and health, Bert Brunekreef, Stephen T Holgate, The Lancet, Volume 360, Issue 9341, 19 October 2002, Pages 1233–1242
6. Department of Environment, Food and Rural Affairs (DEFRA), department website, weblink (accessed 2/12/13):
http://aqma.defra.gov.uk/aqma/local_authorities.php?la_id=296
7. Epidemiology, pathophysiology, and etiology of congestive heart failure in older adults. Rich MW. J Am Geriatr Soc. 1997 Aug;45(8):968-74.
8. Influence of socioeconomic deprivation on the primary care burden and treatment of patients with a diagnosis of heart failure in general practice in Scotland: population based study. F A McAlister, N F Murphy, et al. BMJ. 2004 May 8; 328(7448): 1110.
9. Warwickshire Observatory website – ONS population estimates. Web link (accessed on 3/10/13):
http://www.warwickshireobservatory.org/observatory/observatorywcc.nsf/05a17cfbd3bcf85d802572920033cf0f/bfb8b0a1d34f274c802572c00048108f?OpenDocument
10. Deprivation data produced by the Department for Communities and Local Government. File located at web link (accessed on 3/10/13):
https://www.gov.uk/government/publications/english-indices-of-deprivation-2010
Ward based population weighted averages calculated from LSOA specific data by the London Health Observatory and North East Public Health Observatory.
11. Fahrmeir L, Lang S. Bayesian Inference for Generalized Additive Mixed Models Based on Markov Random Field Priors. *Applied Statistics (JRSS C)*. 2001;50:201-20.
12. Kandala N-B, Tigbe W, Manda S, Stranges S. Spatial Variation of Predictors of Hypertension in Sub-Saharan Africa: A Case Study of South-Africa. American Journal of Hypertension 2013. 10.1093/ajh/hps063.
13. Belitz C, Brezger A, Kneib T, Lang S. BayesX Software for Bayesian Inference in Structured Additive Regression Models Version 2.0.1, 2012. Accessed at:
http://www.stat.uni-muenchen.de/~bayesx/manual/methodology_manual.pdf
14. Taken from the Warwickshire Observatory Quality of Life in Warwickshire 2012/13 report. Weblink (accessed on 3/10/13):
http://maps.warwickshire.gov.uk/iareports/environmental_issues/

For peer review only - http://bmjopen.bmj.com/site/about/guidelines.xhtml

Author's contributions

O B Conceived the idea analyzed the data, contributed to formulating the results and wrote the first draft.

N-B K analyzed the data, advised on statistical aspects, contributed to formulating the results wrote the second draft. CJ analyzed the data. J L helped coordinate the project and co-wrote the final draft; AC coordinated the project, advised on all aspects and co-wrote the final draft.

For peer review only

For peer review only - http://bmjopen.bmj.com/site/about/guidelines.xhtml

Figure legends

Figure 1 : Warwickshire map with 2010 air quality index (all components of air pollution combined) displayed by ward (left; Warwickshire map with number of heart failure hospital admissions per 1000 population between 2005 – 2013 displayed by ward(centre) and Warwickshire map with total heart failure deaths per 1000 population between 2007 – 2012 displayed by ward(right).

Figure 2: Left: Unadjusted total residual spatial effects of morbidity risk associated with heart failure, at ward level in Warwickshire. Shown is the posterior means. Right: Corresponding posterior probabilities at 80% nominal level.

Figure 3: Left: adjusted total residual spatial effects of morbidity risk associated with heart failure, at ward level in Warwickshire. Shown is the posterior means of the full model (IMD 2010, Over 50 and 4 indicators air pollution). Right: Corresponding posterior probabilities at 80% nominal level.

Figure 4: Left: Unadjusted total residual spatial effects of mortality risk associated with heart failure, at ward level in Warwickshire. Shown is the posterior means. Right: Corresponding posterior probabilities at 90% nominal level.

Figure 5: Left: Adjusted total residual spatial effects of mortality risk associated with heart failure, at ward level in Warwickshire. Shown is the posterior means of the full model (IMD 2010, Over 50 and 4 indicators air pollution). Right: Corresponding posterior probabilities at 80% nominal level.

Figure 6 – Warwickshire map displaying the 2010 levels of deprivation (expressed as Multiple Deprivation Scores) by LSOAs. Produced by the Warwickshire Observatory (left) and Warwickshire map displaying the 2008 midyear estimates of % of people over the age of 50 by SOA. Produced by the Warwickshire Observatory (right).

For peer review only

For peer review only - http://bmjopen.bmj.com/site/about/guidelines.xhtml

Table 1: Posterior mean (PM) of fixed effects estimates of heart failure admission and mortality across air pollutions indicators (Warwickshire 2005-2013)

**Variable**	Heart failure admission PM & 95%CI‡	Heart failure mortality PM & 95%CI †
Constant	6.19 (3.84, 8.97)	4.13 (1.18, 7.43)
Nitrogen dioxide indicator (No2)	3.35 (1.89, 4.99)	4.30 (1.68, 7.37)
Sulphur dioxide indicator (So2))	7.75 (-3.84, 17.99)	11.02 (-1.21, 22.41)
Particulates indicator (Pm)	-12.93 (-20.41, -6.54)	-14.69 (-23.46, -6.50)
Benzene indicator (Ben)	[31.9:8.36, 55.85]	25.98 (-6.62, 54.22)
Over 50 years %	0.70(-0.63, 1.98)	1.60, (0.47, 2.92)
IMD 2010 score	0.02 (0.01, 0.03)	0.00 (-0.01, 0.01)

‡ Spatially adjusted posterior mean (PM) from Bayesian geo-additive regression models after controlling for fixed effect of all air pollutions indicators: Mono-nitrogen Oxide (NOx), sulphur dioxide (So2), particulate matter (Pm), benzene (Ben) and combined index and the county of residence (spatial effects). †Spatially adjusted posterior mean (PM) from Bayesian geo-additive regression models after controlling for fixed effect of all air pollutions indicators: Mono-nitrogen Oxide (NOx), sulphur dioxide (So2), particulate matter (Pm), benzene (Ben) and combined index and the county of residence (spatial effects).

For peer review only

For peer review only - http://bmjopen.bmj.com/site/about/guidelines.xhtml

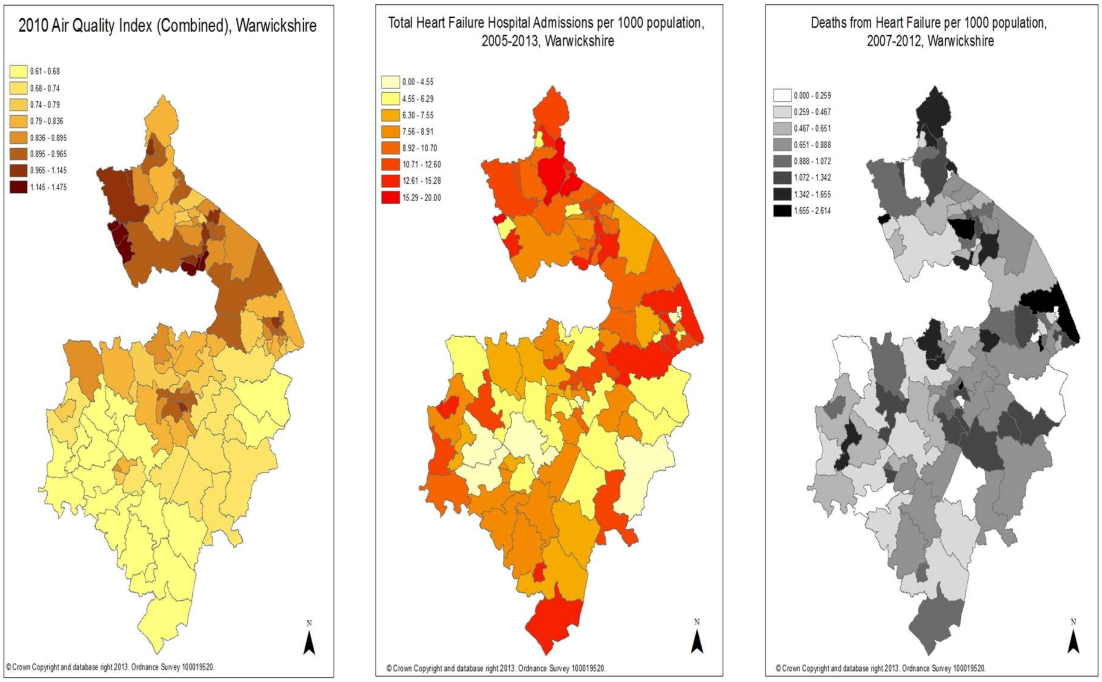

Figure 1 – Warwickshire map with 2010 air quality index (all components of air pollution combined) displayed by ward (left; Warwickshire map with number of heart failure hospital admissions per 1000 population between 2005 – 2013 displayed by ward(centre) and Warwickshire map with total heart failure deaths per 1000 population between 2007 – 2012 displayed by ward(right).

For peer review only

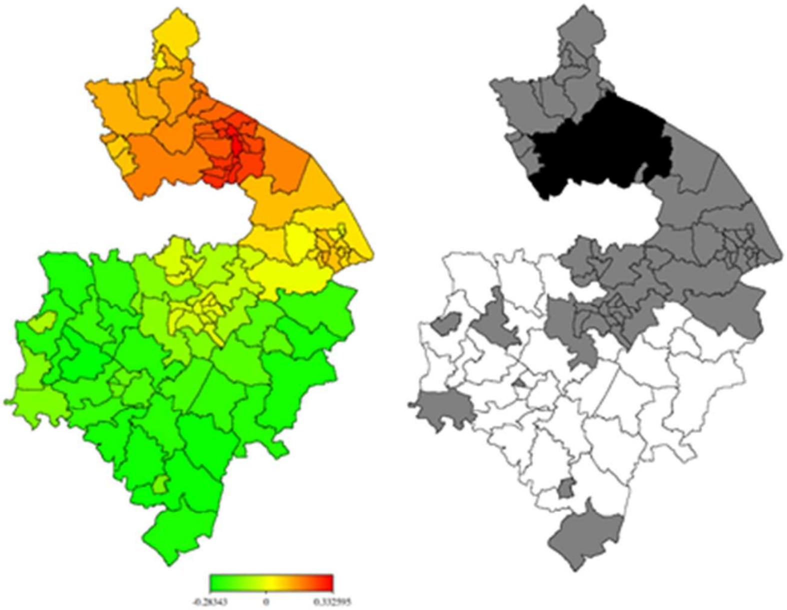

Red coloured – high risk
Green coloured – low risk

Black coloured – significant positive spatial effect
White coloured- significant negative spatial effect
Grey coloured – no significant effect

Figure 2: Left: Unadjusted total residual spatial effects of morbidity risk associated with heart failure, at ward level in Warwickshire. Shown is the posterior means. Right: Corresponding posterior probabilities at 80% nominal level.

For peer review only

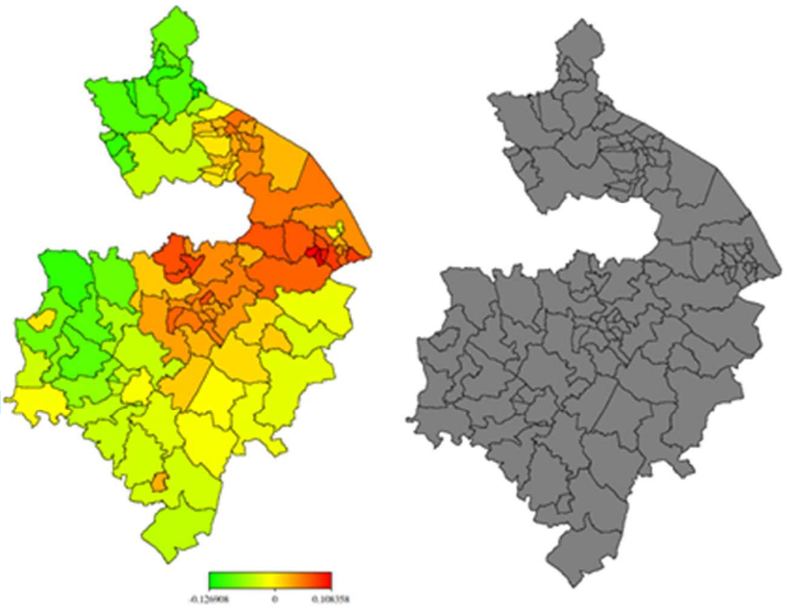

Red coloured – high risk
Green coloured – low risk

Black coloured – significant positive spatial effect
White coloured- significant negative spatial effect
Grey coloured – no significant effect

Figure 3: Left: Total residual spatial effects of morbidity risk associated with heart failure, at ward level in Warwickshire. Shown is the posterior means of the full model (IMD 2010, Over 50 and 4 indicators air pollution). Right: Corresponding posterior probabilities at 80% nominal level.

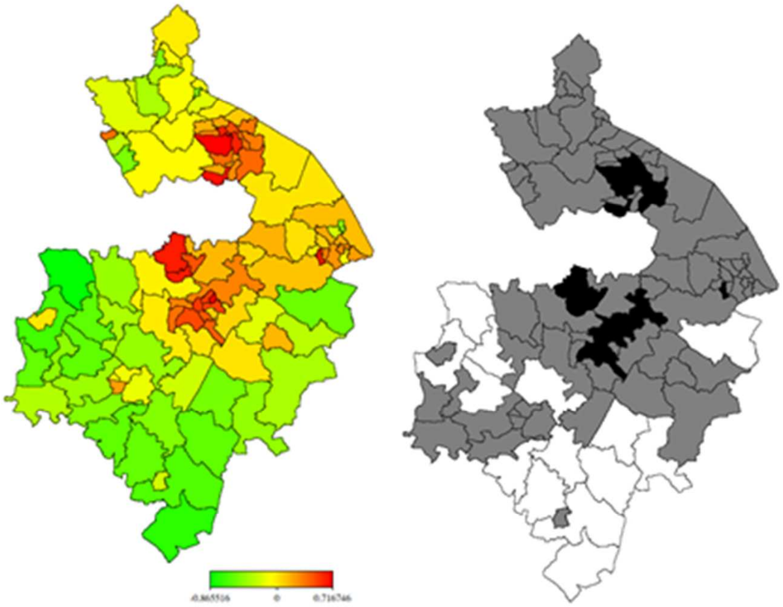

Red coloured – high risk
Green coloured – low risk

Black coloured – significant positive spatial effect
White coloured- significant negative spatial effect
Grey coloured – no significant effect

Figure 4: Left: Unadjusted total residual spatial effects of mortality risk associated with heart failure, at ward level in Warwickshire. Shown is the posterior means. Right: Corresponding posterior probabilities at 80% nominal level.

For peer review only

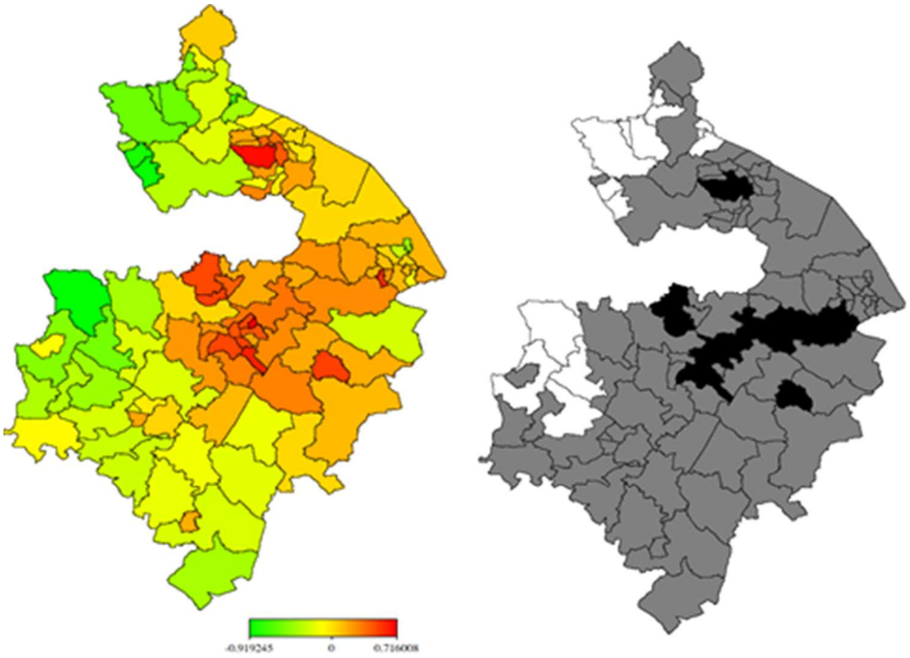

Red coloured – high risk
Green coloured – low risk

Black coloured – significant positive spatial effect
White coloured- significant negative spatial effect
Grey coloured – no significant effect

Figure 5: Left: Total residual spatial effects of mortality risk associated with heart failure, at ward level in Warwickshire. Shown is the posterior means of the full model (IMD 2010, Over 50 and 4 indicators air pollution). Right: Corresponding posterior probabilities at 80% nominal level.

For peer review only

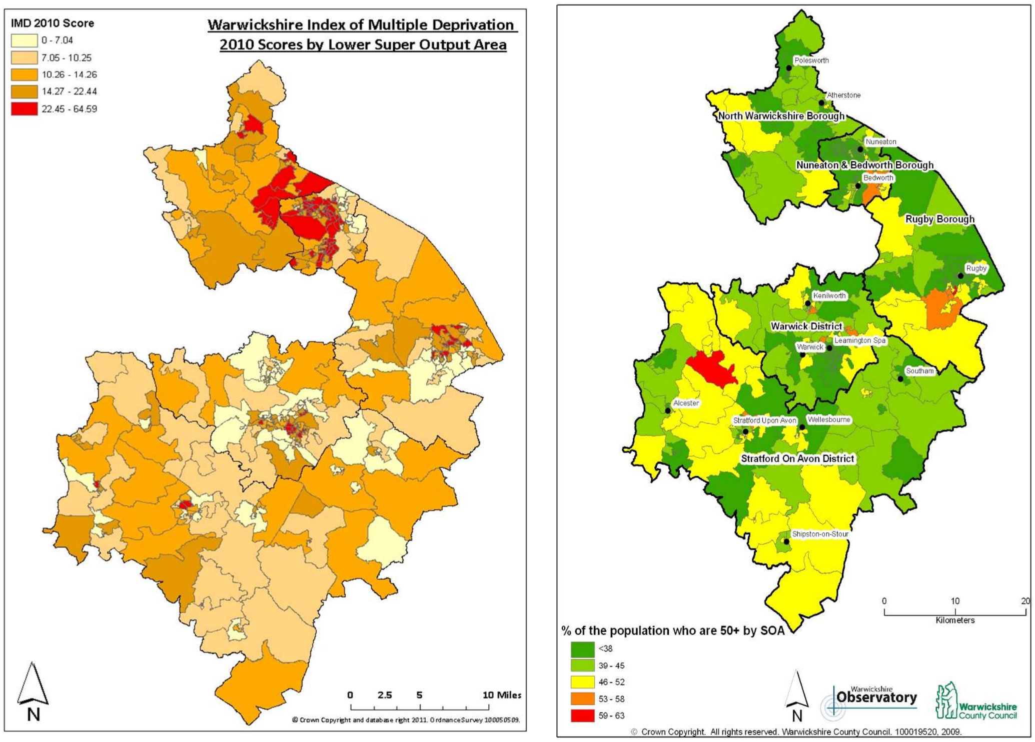

Figure 6 – Warwickshire map displaying the 2010 levels of deprivation (expressed as Multiple Deprivation Scores) by LSOAs. Produced by the Warwickshire Observatory (left) and Warwickshire map displaying the 2008 midyear estimates of % of people over the age of 50 by SOA. Produced by the Warwickshire Observatory (right).

For peer review only

STROBE 2007 (v4) Statement—Checklist of items that should be included in reports of *cross-sectional studies*

Section/Topic	Item #	Recommendation	Reported on page #
**Title and abstract**	1	(*a*) Indicate the study's design with a commonly used term in the title or the abstract	2
		(*b*) Provide in the abstract an informative and balanced summary of what was done and what was found	2
**Introduction**			
Background/rationale	2	Explain the scientific background and rationale for the investigation being reported	4
Objectives	3	State specific objectives, including any prespecified hypotheses	4,5
**Methods**			
Study design	4	Present key elements of study design early in the paper	4, 5
Setting	5	Describe the setting, locations, and relevant dates, including periods of recruitment, exposure, follow-up, and data collection	5,6
Participants	6	(*a*) Give the eligibility criteria, and the sources and methods of selection of participants	5,6,
Variables	7	Clearly define all outcomes, exposures, predictors, potential confounders, and effect modifiers. Give diagnostic criteria, if applicable	5-7
Data sources/ measurement	8*	For each variable of interest, give sources of data and details of methods of assessment (measurement). Describe comparability of assessment methods if there is more than one group	5-6
Bias	9	Describe any efforts to address potential sources of bias	*7-8,12*
Study size	10	Explain how the study size was arrived at	5
Quantitative variables	11	Explain how quantitative variables were handled in the analyses. If applicable, describe which groupings were chosen and why	7-8
Statistical methods	12	(*a*) Describe all statistical methods, including those used to control for confounding	7,8
		(*b*) Describe any methods used to examine subgroups and interactions	NA
		(*c*) Explain how missing data were addressed	NA
		(*d*) If applicable, describe analytical methods taking account of sampling strategy	NA
		(*e*) Describe any sensitivity analyses	NA
**Results**			

For peer review only

Participants	13*	(a) Report numbers of individuals at each stage of study—eg numbers potentially eligible, examined for eligibility, confirmed eligible, included in the study, completing follow-up, and analysed	2, 5,6
		(b) Give reasons for non-participation at each stage	NA
		(c) Consider use of a flow diagram	NA
Descriptive data	14*	(a) Give characteristics of study participants (eg demographic, clinical, social) and information on exposures and potential confounders	6
		(b) Indicate number of participants with missing data for each variable of interest	NA
Outcome data	15*	Report numbers of outcome events or summary measures	10-11
Main results	16	(*a*) Give unadjusted estimates and, if applicable, confounder-adjusted estimates and their precision (eg, 95% confidence interval). Make clear which confounders were adjusted for and why they were included	8
		(*b*) Report category boundaries when continuous variables were categorized	NA
		(*c*) If relevant, consider translating estimates of relative risk into absolute risk for a meaningful time period	NA
Other analyses	17	Report other analyses done—eg analyses of subgroups and interactions, and sensitivity analyses	NA
**Discussion**			
Key results	18	Summarise key results with reference to study objectives	8-10
Limitations	19	Discuss limitations of the study, taking into account sources of potential bias or imprecision. Discuss both direction and magnitude of any potential bias	11, 15
Interpretation	20	Give a cautious overall interpretation of results considering objectives, limitations, multiplicity of analyses, results from similar studies, and other relevant evidence	11-15
Generalisability	21	Discuss the generalisability (external validity) of the study results	11-15
**Other information**			
Funding	22	Give the source of funding and the role of the funders for the present study and, if applicable, for the original study on which the present article is based	NA

*Give information separately for cases and controls in case-control studies and, if applicable, for exposed and unexposed groups in cohort and cross-sectional studies.

Note: An Explanation and Elaboration article discusses each checklist item and gives methodological background and published examples of transparent reporting. The STROBE checklist is best used in conjunction with this article (freely available on the Web sites of PLoS Medicine at http://www.plosmedicine.org/, Annals of Internal Medicine at http://www.annals.org/, and Epidemiology at http://www.epidem.com/). Information on the STROBE Initiative is available at www.strobe-statement.org.

For peer review only

BMJ Open

Spatial variation of Heart Failure and Air Pollution in Warwickshire, UK: An investigation of small scale variation at the ward-level

Journal:	*BMJ Open*
Manuscript ID:	bmjopen-2014-006028.R1
Article Type:	Research
Date Submitted by the Author:	19-Aug-2014
Complete List of Authors:	Bennett, Oscar; Warwickshire County Council, Public Health Warwickshire,Communities Group<br>Kandala, Ngianga-Bakwin; University of Warwick, Warwick Medical School<br>Ji, Chen; University of Warwick, Warwick Medical School<br>Linnane, John; Warwickshire County Council, Public Health Warwickshire,Communities Group<br>Clarke, Aileen; University of Warwick, Division of Health Sciences
**Primary Subject Heading**:	Epidemiology
Secondary Subject Heading:	Cardiovascular medicine, Epidemiology, Public health
Keywords:	Heart failure < CARDIOLOGY, EPIDEMIOLOGY, SOCIAL MEDICINE

SCHOLARONE™ Manuscripts

For peer review only

Spatial variation of Heart Failure and Air Pollution in Warwickshire, UK: An investigation of small scale variation at the ward-level

Oscar Bennett MB BChir[1], *Ngianga-Bakwin Kandala PhD*[2, 3], *Chen Ji PhD*[2], *John Linnane MD* FFPHM[1], *Aileen Clarke MD PhD*[2]

[1] Public Health Warwickshire, Communities Group, Warwickshire County Council ,Shire Hall, Barrack Street, Warwick, CV34 4RL, UK.
[2] Division of Health Sciences, University of Warwick Medical School, Coventry, CV4 7AL, UK.
[3] Division of Epidemiology and Biostatistics, School of Public Health, University of Witwatersrand, Johannesburg , South Africa.

Running title: Heart Failure and Air Pollution in Warwickshire

Keywords: Warwickshire, Heart Failure, Air Pollution, epidemiology, spatial analyses

Correspondence to:

Ngianga-Bakwin Kandala, PhD
Division of Health Sciences
University of Warwick Medical School
Medical School Building
Gibbet Hill Campus
Coventry CV4 7AL
United Kingdom
Tel: + 44 (0) 2476150541
Fax:+ 44 (0) 2476528375
Email: N-B.Kandala@warwick.ac.uk

For peer review only

For peer review only - http://bmjopen.bmj.com/site/about/guidelines.xhtml

Abstract

Objectives: To spatially map the morbidity and mortality caused by heart failure within Warwickshire to characterise and quantify any influence of air pollution on these risks.

Design: Cross-sectional.

Setting: Warwickshire, United Kingdom

Participants: Data from all of the 105 current Warwickshire County wards were collected on hospital admissions and deaths due to heart failure.

Results

In multivariate analyses, the presence of a higher NOx in a ward [3.35:1.89, 4.99], Ben [31.9:8.36, 55.85] and IMD [0.02: 0.01, 0.03], were consistently associated with a higher risk of heart failure morbidity. Pm [-12.93:-20.41, -6.54] was negatively associated with the risk of heart failure morbidity. No association was found between So2 and heart failure morbidity.
The risk of heart failure mortality was higher in wards with a higher NOx [4.30:1.68, 7.37] and wards with more inhabitants 50+ years old [1.60: 0.47, 2.92]. Pm was negatively associated [-14.69: -23.46, -6.50] with heart failure mortality. So2, Ben, and IMD score were not associated with heart failure mortality.
There was a prominent variation in heart failure morbidity and mortality risk across wards, the highest risk being in the regions around Nuneaton and Bedworth.

Conclusion

This study showed distinct spatial patterns in heart failure morbidity and mortality, suggesting the potential role of environmental factors beyond individual-level risk factors. Air pollution levels should therefore be taken into account when considering the wider determinants of public health and the impact that changes in air pollution might have on the health of a population.

For peer review only - http://bmjopen.bmj.com/site/about/guidelines.xhtml

ARTICLE FOCUS

- Air pollution has been linked to the development and exacerbation of a number of health problems including cardiovascular diseases such as heart failure.
- Heart failure is a serious condition that unfortunately affects many people in the UK. This link seems especially clear for cardiovascular and respiratory diseases such as ischaemic heart disease, heart failure, asthma, influenza, and lung cancer.
- It is well established that the ambient levels of air pollution in a region can have an impact on the health status of the population which inhabits it

KEY MESSAGES

1. Using a novel approach, this study has characterised and quantified the potential role of environmental factors (air pollutants) at the county-level.
2. The presence of a higher Mono-nitrogen Oxide (NOx) in a ward, benzene and poor Index of Multiple Deprivation (IMD) score were consistently associated with a higher risk of heart failure morbidity. Particulate matter (Pm) was negatively associated with the risk of heart failure morbidity.
3. The risk of heart failure mortality was higher in wards with a higher NOx and wards with more inhabitants 50+ years old. Pm was negatively associated with heart failure mortality.
4. Air pollution overall, when all pollution components were brought together into a combined index, was positively associated with both heart failure morbidity and mortality across Warwickshire.
5. There was a striking variation in heart failure morbidity and mortality risk across wards, the highest risk being in the regions around Nuneaton and Bedworth.

STRENGTHS AND LIMITATIONS

- The model employed a fully Bayesian approach using Markov Chain Monte Carlo (MCMC) techniques for inference and model checking [11, 12]

- A single air pollution measurement in 2010 was used and it was assumed that there was no significant change in this value over the 2005-2013 periods that mortality and hospital admission data was gathered from.
- The cross-sectional nature of the study unfortunately does not allow us to establish temporality and thus clearly demonstrate causality of the observed associations.
- This was an ecological study, analysing characteristics and risk at the ward rather than individual level. Consequently associations and conclusions found from the aggregate data may not be directly applicable to individuals.

For peer review only - http://bmjopen.bmj.com/site/about/guidelines.xhtml

Introduction

Air pollution has been linked to the development and exacerbation of a number of health problems. This link seems especially clear for cardiovascular and respiratory diseases such as ischaemic heart disease, heart failure, asthma, influenza, and lung cancer [1, 2, 3, 4]. It is well established that the ambient levels of air pollution in a region can have an impact on the health status of the population which inhabits it [5]. Air pollution levels should therefore be taken into account when considering the wider determinants of public health and the impact that changes in air pollution might have on the health of a population.

Warwickshire is an English county within the United Kingdom approximately 70 miles North-West of London. The issue of air pollution has been highlighted in Warwickshire recently by the setting up of Air Quality Management Areas (AQMAs) in a number of different parts of the county. These are specific areas in the county that have been identified as places where, without a focused local council strategy to reduce air pollution, future government targets for air pollutant concentrations may not be met. Within Warwickshire the specific air pollutant that has been identified as a problem and led to these special areas being setup is mono-nitrogen oxides (NOx)[6].

The objective of the present study was therefore to look for any relationship between levels of air pollution and the morbidity and mortality associated with heart failure within Warwickshire in the last few years (2005-2013 for morbidity, 2007-2012 for mortality). We examined a range of traditional air pollutants for an ecological association with heart failure morbidity and mortality at the county level. Furthermore, the present analysis attempted to highlight spatial patterns in heart failure morbidity and mortality risk within the county, after multiple adjustments for proximate, county-level factors. The geographic locations of wards within the county can be considered as proxy measures of many other unmeasured factors such as availability and access to health services, individual health seeking behaviour,

For peer review only - http://bmjopen.bmj.com/site/about/guidelines.xhtml

preventive ward policy and general ward factors. Such estimates might illustrate how much can be learned by detailed exploratory analyses as well as how these data can be used to strategically inform policy aiming at the prevention and management of serious conditions such as heart failure in these settings.

The evidence from published literature for the link between air pollution and health problems

Studies have shown an effect on the health of populations caused by both long term exposure as well as short term "spikes" in local air pollution levels[5].

A relevant systematic review and meta-analysis has been published recently in the Lancet [2]. It pooled the results of studies that have been conducted worldwide looking at the temporal relationship between the levels of a number of different air pollutants with local heart failure hospital admission rates and mortality rates. This showed a clear link between short term rises in all types of air pollution (except ozone) and rises in hospital admissions and mortality due to heart failure. This study did not look at any potential effects from long term exposure to air pollution. However it did provide strong evidence that air pollution can 'exacerbate' heart failure, increasing the likelihood that a patient with existing heart failure will become sufficiently unwell to require hospital admission, or even die.

The ESCAPE study[3], published recently in the Lancet Oncology, pooling the results of 17 cohort studies from around Europe looked at the ambient levels of air pollution in an area (particulate matter and NOx) and the incidence of lung cancers in the inhabitants of this area during many years of follow up (mean 12.8 years). This was intended to look at the risk associated with long term exposure to air pollution. A statistically significant correlation was found between the levels of particulate matter air pollution and the local incidence of lung cancer diagnoses. Particulate matter with a diameter of less than 10 µm had a hazard ratio of 1.22 [95% CI 1.03-1.45] per (10 µg/cubic meter). In other words, for every increase in

For peer review only - http://bmjopen.bmj.com/site/about/guidelines.xhtml

particulate matter pollution of 10 μg/cubic meter there was a corresponding immediate increase in the chance of being diagnosed with lung cancer of 22% (95% CI 3% - 45%). There was also a correlation between traffic volume within 100m of a residence and a modest increase in the rate of lung cancer (Hazard ratio 1.09 (CI 0.99-1.21)). No similar correlation was found with NOx air pollution. This suggests that long term exposure to higher levels of particulate matter air pollution may increase the incidence of lung cancer in a population. Another study published in the United States in 2004 looked at the long term effect of exposure to particulate matter air pollution and the mortality attributed to different cardiovascular and respiratory diseases in different areas of the United States[(4)]. A good correlation was found between the degree of long term exposure to particulate matter air pollution and increases in mortality from cardiovascular diseases, including heart failure. Interestingly this was not found to be the case for most respiratory diseases. There was also an element of the study that looked at the effect of a person's smoking status on the mortality statistics. This found, as expected, a strong link between mortality and smoking. However it also found that air pollution contributed additional cardiovascular mortality risk on top of that attributed to smoking. This was at least an additive, if not a synergistic effect.

Methods

Study data

In order to carry out this project, data was collected about the geographical distribution of air pollution within Warwickshire. This data included information about each of four individual components of air pollution (NOx, sulphur dioxide, particulate matter, and benzene) which could then be united into a combined index (all of the contributions added together). A single recorded level from 2010 of each air pollutant for each ward was used in the study. This was then compared to collected data about:

For peer review only - http://bmjopen.bmj.com/site/about/guidelines.xhtml

(i) The geographical distribution of home addresses of patients who were admitted to hospital because of heart failure or a complication of heart failure. Hospital admission rate in an area due to heart failure was used as a proxy indicator for the level of heart failure morbidity within that area.

(ii) The geographical distribution of home addresses of patients who died from heart failure, or whose death was contributed to by heart failure.

These data were collected by the Warwickshire Observatory, which is part of the Warwickshire County Council in charge of collecting and handling statistics relating to the county. Mortality data for the analysis was supplied via the Warwickshire Public Health Intelligence Team and was sourced from the Public Health Mortality Files, Office for National Statistics. Hospital admissions data was accessed via the Ventris Business Intelligence System, Arden Commissioning Support Unit."

Ward level population data were obtained from the 2011 census. Warwickshire is divided into 105 wards. Data from all of the 105 current Warwickshire County wards were collected. The potential confounding variables of firstly age structure of a population within a ward and secondly levels of social deprivation within a ward were identified [7,8]. These were then controlled for in the second stage of the statistical analysis (see below).

Information about the age structure of wards was obtained from the Office of National Statistics mid 2010 estimates (obtained from the Warwickshire Observatory website)[9]. The representative statistical value of "percentage (%) of population above age 50" was used as an indicator of wards with a higher proportion of older people.

Information about social deprivation was obtained from the English Indices of Deprivation published by the Department for Communities and Local Government [10]. The Index of Multiple Deprivation (IMD) averaged across each ward was used as an indicator of the level of deprivation within the wards of Warwickshire.

For peer review only - http://bmjopen.bmj.com/site/about/guidelines.xhtml

The information on crude observed air pollution level distribution, heart failure hospital admission rates and mortality rates were then represented on maps of Warwickshire (**Figure 1**).

Statistical Analysis

To account for spatial autocorrelation in observed heart failure hospital admission and mortality rates at the ward level, in Warwickshire, we applied a unified approach to account for possible air pollution effects of environmental risk factors. This was achieved using a geo-additive semi-parametric mixed model. The model employed a fully Bayesian approach using Markov Chain Monte Carlo (MCMC) techniques for inference and model checking [11, 12]. Response variables were defined as the count (per 1000 population) of heart failure morbidity or mortality in a ward (Poisson model): $y_i / \eta_i, \delta \sim \mathrm{B}(\eta_i, \delta)$ for a binomial formulation.

(Poisson model): $y_i / \eta_i, \delta \sim \mathrm{B}(\eta_i, \delta)$ (1) for a binomial formulation and a geo-additive semi-parametric predictor $\mu_i = h(\eta_i)$:

$$\eta_i = f1(xi1) + ... + f_{p(xip)} + f_{spat}(s_i) + \varepsilon_i \qquad (2)$$

where h is a known response function with a poison link function, $f_1, ..., f_p$ are non-linear smoothed effects of the metrical covariates (time in years), and $f_{spat}(s_i)$ is the effect of the spatial covariate $s_i \in \{1, ..., S\}$ labelling the ward in Warwickshire. Regression models with predictors such as those in equation 2 are sometimes referred to as geo-additive models. P-spline priors were assigned to the functions $f1, ..., f_p$, and a Markov random field prior was used for $f_{spat}(s_i)$. More detailed information about the modelling approach can be found elsewhere [11, 13]. The standard measure of effect was the posterior mean (PM) and 95% credible region (CR).

The analysis was carried out using version 2.0.1 of the BayesX software package, which permits Bayesian inference based on MCMC simulation techniques[13]. Multivariate Bayesian geo-additive regression models were used to evaluate the significance of the posterior mean

For peer review only - http://bmjopen.bmj.com/site/about/guidelines.xhtml

(PM) determined for the fixed effects and spatial effects between air pollution and the morbidity and mortality from heart failure within Warwickshire. Each component of air pollution was looked at separately and then also the combined index in unadjusted models. Next, fully adjusted multivariate Bayesian geo-additive regressions analysis were performed to look again for statistically significant correlation between these variables, but this time further controlling for any influence from age structure or social deprivation.

Results

Figure 1 and **Figure 6** display the observed data collected for this study on maps using graduated colouring to represent data value categories within each ward.

Observed Air Quality Map

Figure 1left was produced using 2010 data from the Warwickshire Observatory. It is based on a combined air quality indicator which is a combination of information about the contribution to air pollution from NOx, sulphur dioxide, particulate matter, and benzene. The Warwickshire Observatory description of this index reads as follows:

"Combined Air Quality Indicator (estimates of emissions for four pollutants: benzene, nitrogen dioxide, sulphur dioxide and particulates) for small areas (modelled to 1 km grid squares) where an index value of 1 is equivalent to the national standard for each pollutant. The values are then summed so an overall score of 4 would represent all four pollutants being present at the national standard level."[(14)] These specific standards are described in the Air Quality Standards Regulations 2010.

The geographical pattern of the observed air pollution across the county shows a higher level of air pollution near the more urban centres of Birmingham and Coventry. The proximity of parts of the county to motorways such as the M6 and M42 could also be a contributing factor

For peer review only - http://bmjopen.bmj.com/site/about/guidelines.xhtml

For peer review only

to the observed pattern. There was no place in the county where the level of any pollutant exceeded the national standard in 2010.

Heart Failure Hospital Admission Map

Figure 1centre shows the geographical distribution of the density of home addresses of patients admitted to hospital with a diagnosis of heart failure (or exacerbation of heart failure) within the April 2005- April 2013 period.

Heart Failure Mortality Map

Figure 1right shows the geographical distribution of the density of home addresses of patients who died either directly or in part from heart failure in the 2007-2012 (inclusive) periods.

Table 1 (left panel) displays posterior means of heart failure admission across the selected covariates following multivariate Bayesian geo-additive regression analyses.

The Posterior mean (PM) & 95% Credible Region (CR) of overall hospital admission rates due to heart failure and mortality rates from heart failure were [6.19 (3.84, 8.97)] and [4.13 (1.18, 7.43)] (Table 2) respectively. On average, the presence of a higher Mono-nitrogen oxide indicator (NOx) in a ward [Posterior mean (PM) & 95% Credible Region (CR): 3.35 (1.89, 4.99)], Benzene indicator (Ben) [31.9 (8.36, 55.85)] and IMD 2010 score [0.02 (0.01, 0.03)], were consistently associated with higher risk of heart failure morbidity. The particulates indicator (Pm) [-12.93 (-20.41, -6.54)] was negatively associated with the risk of heart failure morbidity. Sulphur dioxide indicator (So2) was not associated with heart failure morbidity.

For peer review only - http://bmjopen.bmj.com/site/about/guidelines.xhtml

Table 1 (right panel) shows the same corresponding results for heart failure mortality. This table shows that the risk of heart failure mortality was higher in wards with a higher Mono-nitrogen oxide indicator (NOx) [4.30 (1.68, 7.37)] and wards with more inhabitants over 50 years old [1.60 (0.47, 2.92)]. The particulates indicator (Pm) was negatively associated with heart failure mortality. The sulphur dioxide indicator (So2), Benzene indicator (Ben), and IMD score were not associated with heart failure mortality.

The combined air pollution index (all indicators averaged together) when incorporated into a separate model combining age and social deprivation was significantly positively associated with both heart failure morbidity [1.39 (0.87, 1.81)] and mortality [1.79 (0.85, 2.55)] across the county.

In **Figure 2** and **Figure 4**, the left-hand maps show the unadjusted estimates of posterior total residual ward means of heart failure morbidity and mortality respectively. In **Figure 3** and **Figure 5**, the left-hand maps show the adjusted PM after multiple adjustment for the geographical location, taking into account the auto-correlation structure in the data, the uncertainty in the ward level and all ward-level risk factors (air pollution components, age, social deprivation) for heart failure morbidity and mortality respectively. The red colour indicates the maximum posterior mean recorded while green denotes the lowest mean. The right-hand maps in **Figures 2**, **3, 4** and **5** show the 95% posterior probability of heart failure and mortality, which indicate the statistical significance associated with the total excess risk. Black colour indicates a negative spatial effect (associated with increased risk of heart failure and mortality), white colour a positive effect (a decreased risk) and grey colour a non-significant effect.

In general, there was consistently higher heart failure morbidity risk in northern wards particularly around Nuneaton and Bedworth and lower heart failure morbidity risks in the

For peer review only - http://bmjopen.bmj.com/site/about/guidelines.xhtml

southern wards particularly within the district of Stratford-on-Avon. However, all this variation could partially be accounted for within the model generated within this study (taking into account air pollution, age, and social deprivation levels).

Heart failure mortality risk was, in general, higher in northern wards around Nuneaton and Bedworth as well as in some central areas around Warwick, Royal Leamington Spa, and Kenilworth. Heart failure mortality risk was again lower in more southern wards particularly within Stratford-on-Avon. Unlike with morbidity however, our model could not explain all this variation in heart failure mortality. Even after taking into account air pollution, age, and social deprivation the rate of death from heart failure remained significantly higher than would be expected in areas within and around Nuneaton, Bedworth, Warwick, Royal Leamington Spa, and Kenilworth.

In sensitivity analyses, we tested several models and our results were not substantially altered after removing one or two pollutant (data not shown).

Discussion

Before even considering air pollution, this study helps to demonstrate the inequality of risk from heart failure disease and death that exists for individuals living in different parts of the county of Warwickshire. There is a significant excess risk of both disease and death in more northern wards within and surrounding Nuneaton and Bedworth. Much (but not all) of this variation could be attributed to the air pollution, age structure and social deprivation that exists in these areas according to our model. Measures that seek to address air pollution and social deprivation could be expected therefore to help mitigate against cardiovascular risks within these local populations.

For peer review only - http://bmjopen.bmj.com/site/about/guidelines.xhtml

The present study corroborates the notion that air pollution is an increasingly important public health issue in Warwickshire. Higher levels of the average air pollution index looked at in this study correlated significantly with increased levels of both heart failure morbidity and mortality across the county, even after removing the effects of age structure and social deprivation. The individual component of NOx air pollution seems particularly to contribute risk to both the morbidity and mortality of heart failure within the county, suggesting that it may have a particularly detrimental effect on heart failure patients. This reinforces the importance of the AQMAs set up within the county in response to high NOx levels. Road traffic is a large contributor to air pollution within Warwickshire. Diesel engines are responsible for a large part of the NOx component of these emissions.

An unexpected result also appeared within our analysis. Particulate matter air pollution became significantly, negatively correlated with both heart failure morbidity and mortality when incorporated into our model with all risk factors taken into account and controlled for in this study. This would imply some sort of unexpected "protective influence" from particulate matter air pollution on heart failure patients. This clearly contradicts our expectations and is at odds with a wealth of existing evidence that indicates that particulate matter air pollution contributes risk to and exacerbates cardiovascular disease[(4)].

We offer a possible explanation for this based on the following four observations:

1. The above-mentioned negative correlation of particulate matter air pollution with heart failure morbidity and mortality in our model.
2. Particulate matter air pollution actually varied very little across the county compared to the other types of air pollution. All types of air pollution tended to decrease in rural areas, but particulate matter tended to decrease much *less* compared to the other components of air pollution. Consequently in rural areas of the county where most

For peer review only - http://bmjopen.bmj.com/site/about/guidelines.xhtml

types of air pollution are significantly lower, particulate matter pollution was relatively higher compared to NOx, Benzene, and SO2.

3. There seems to be a high risk of heart failure deaths in urban centres (particularly Nuneaton, Bedworth, Warwick, Royal Leamington Spa, and Kenilworth), higher than can be explained by our model.
4. Conversely, there seems to be a particularly low risk of heart failure deaths in some rural areas within the western part of Stratford-on-Avon, lower than can be explained by our model.

A possible hypothesis based on these observations is that there is an additional factor influencing the morbidity and mortality of heart failure not looked at in this study, namely the urban/rural nature of a patient's living environment. It could be the case that living in an urban environment contributed risk and living in a rural area provided protection against heart failure morbidity and mortality. This would be an effect in *addition* to any increase in air pollution or social deprivation within urban settings compared to rural settings. This could certainly in principle be plausible, with people in rural areas perhaps doing more physical activity, eating more healthily etc. If this were the case it would explain the excess deaths in urban centres found in this study. It could also be responsible for the unexpected protective factor attributed to particulate matter air pollution in our analysis. Given that the particulate matter component of air pollution is *relatively* higher than the other components in rural areas, the protection that living in rural areas affords individuals could be misleadingly attributed (in our statistical analysis) to the particulate matter component of air pollution present in these areas. Further work will need to be done looking into this possible link between urban/rural living environments and heart failure morbidity and mortality. It could be very revealing to carefully characterise this effect if it indeed exists, as it may be an indication of unrecognised cardiovascular risk/protective factors associated with urban/rural living that exists within Warwickshire.

For peer review only - http://bmjopen.bmj.com/site/about/guidelines.xhtml

However, it is also important to bear in mind that this is an ecological study and all the relationships picked up between variables in this study have been found using aggregate data at the ward level (number over 50 years of age, average IMD score, average air pollution across ward, overall numbers of deaths and hospital admissions due to heart failure). It is not always a trivial task to extrapolate the conclusions drawn from such a study down to the level of individuals. Such a task would involve drilling down to individual level data and repeating the analysis, a task that was beyond the scope of this particular study. It is certainly possible that the unexpected negative correlation between particulate matter air pollution and heart failure could disappear when data is analysed at the individual level – an example of an ecological fallacy. Consequently it would be prudent to regard the results from the individual components of air pollution with cautious interest, rather than viewing them as proof of any real effects.

However, despite these caveats, this study has been able to provide some helpful information at the population level worthy of consideration. A health inequality has been revealed, and the manner with which this inequality is influenced by age, social deprivation, and the combined index of air pollution has been demonstrated. Such information should help inform policy decisions that would influence society at a population level and hopefully improve public health in the long run.

There are some limitations in this study worth considering that result from assumptions made along the way. A single air pollution measurement in 2010 was used and it was assumed that there was no significant change in this value over the 2005-2013 periods that mortality and hospital admission data was gathered from. The resulting cross-sectional nature of the study does not allow establishing temporality and thus causality of the observed associations. There was also no way to determine the length of time that individual members of the population within a ward had lived in that area, and thus how long they had been exposed to the

For peer review only - http://bmjopen.bmj.com/site/about/guidelines.xhtml

measured ambient air pollution level. It was assumed that people with home addresses in a ward were exposed significantly to the levels of air pollution in that ward. Finally, as already mentioned, this was an ecological study, using aggregate data of risk factors to look for associations with aggregate data of morbidity and mortality. It is not always possible to apply such associations from a population level down to the individuals within that population.

In summary, this study has provided a number of interesting results. Firstly it has helped to quantify and map the inequality that exists across different parts of Warwickshire with regards to heart failure risk. It has also provided some interesting circumstantial evidence of a link between heart failure morbidity and air pollution. Finally it has also given a suggestion of a possible link between living in urban environments and a higher risk of cardiovascular disease, and a corresponding lower risk from living in rural environments. More work will need to be done to look into this particular possibility. It would be informative to run this type of analysis whilst factoring in the influence of a person's distance from their nearest urban centre. This urban/rural factor should be further explored and mined for additional information as it could be an indication of hitherto unconsidered factors influencing the health status of the population of Warwickshire and possibly further afield.

In order to determine the validity of our conclusions at the individual level further work would need to be done analysing the available data from individual patients (risks and outcomes). Such work could help to characterise the true effect of different components of air pollution at the individual level. It would also be interesting to determine if the different components of air pollution act as effect modifiers on each other.

It would be possible to look at the effects of air pollution variation in the shorter term as well. For example, looking at how local "spikes" in air pollution affect the rates of hospital admissions locally immediately following it. This could be done in Leamington Spa where there is an air quality monitoring station constantly measuring the levels of air pollutants.

For peer review only - http://bmjopen.bmj.com/site/about/guidelines.xhtml
For peer review only

Other health problems, such as ischaemic heart disease and respiratory diseases, have been linked with air pollution as well and it could be informative to also look into these links locally.

Contributorship statement:

OB conceived the idea, analyzed the data, contributed to formulating the results and wrote the first draft. N-B K analyzed the data, advised on statistical aspects, contributed to formulating the results, wrote the second draft. CJ analyzed the data. JL helped coordinate the project and co-wrote the final draft; AC coordinated the project, advised on all aspects and co-wrote the final draft.

Funding:

This paper presents independent research supported by the National Institute for Health Research (NIHR) Collaborations for Leadership in Applied Health Research and Care West Midlands. The views expressed are those of the author(s) and not necessarily those of the NHS, the NIHR or the Department of Health.

Competing interests: The authors declared no conflict of interest.

Data sharing: No additional data available

For peer review only

References

1. Particulate Air Pollution and Hospital Emergency Room Visits for Asthma in Seattle, Joel Schwartz, Daniel Slater, Timothy V. Larson, William E. Pierson, and Jane Q. Koenig. American Review of Respiratory Disease, Vol. 147, No. 4 (1993), pp. 826-831.
2. Global association of air pollution and heart failure: a systematic review and meta-analysis, Anoop SV Shah, Jeremy P Langrish,Harish Nair,David A McAllister, Amanda L Hunter, Prof Ken Donaldson, Prof David E Newby, Dr Nicholas L Mills. The Lancet, Early Online Publication, 10 July 2013
DOI: 10.1016/S0140-6736(13)60898-3
3. Air pollution and lung cancer incidence in 17 European cohorts: prospective analyses from the European Study of Cohorts for Air Pollution Effects (ESCAPE), Raaschou-Nielsen O, Andersen ZJ, et al. Lancet Oncol. 2013 Aug;14(9):813-22
4. Cardiovascular Mortality and Long-Term Exposure to Particulate Air Pollution, Epidemiological Evidence of General Pathophysiological Pathways of Disease
C. Arden Pope III, Richard T. Burnett, et al. Circulation. 2004; 109: 71-77
5. Air pollution and health, Bert Brunekreef, Stephen T Holgate, The Lancet, Volume 360, Issue 9341, 19 October 2002, Pages 1233–1242
6. Department of Environment, Food and Rural Affairs (DEFRA), department website, weblink (accessed 2/12/13):
http://aqma.defra.gov.uk/aqma/local_authorities.php?la_id=296
7. Epidemiology, pathophysiology, and etiology of congestive heart failure in older adults. Rich MW. J Am Geriatr Soc. 1997 Aug;45(8):968-74.
8. Influence of socioeconomic deprivation on the primary care burden and treatment of patients with a diagnosis of heart failure in general practice in Scotland: population based study. F A McAlister, N F Murphy, et al. BMJ. 2004 May 8; 328(7448): 1110.
9. Warwickshire Observatory website – ONS population estimates. Web link (accessed on 3/10/13):
http://www.warwickshireobservatory.org/observatory/observatorywcc.nsf/05a17cfbd3bcf85d802572920033cf0f/bfb8b0a1d34f274c802572c00048108f?OpenDocument
10. Deprivation data produced by the Department for Communities and Local Government. File located at web link (accessed on 3/10/13):
https://www.gov.uk/government/publications/english-indices-of-deprivation-2010
Ward based population weighted averages calculated from LSOA specific data by the London Health Observatory and North East Public Health Observatory.
11. Fahrmeir L, Lang S. Bayesian Inference for Generalized Additive Mixed Models Based on Markov Random Field Priors. *Applied Statistics (JRSS C)*. 2001;50:201-20.
12. Kandala N-B, Tigbe W, Manda S, et al. Spatial Variation of Predictors of Hypertension in Sub-Saharan Africa: A Case Study of South-Africa. American Journal of Hypertension 2013. 10.1093/ajh/hps063.
13. Belitz C, Brezger A, Kneib T, et al. BayesX Software for Bayesian Inference in Structured Additive Regression Models Version 2.0.1, 2012. Accessed at:
http://www.stat.uni-muenchen.de/~bayesx/manual/methodology_manual.pdf
14. Taken from the Warwickshire Observatory Quality of Life in Warwickshire 2012/13 report. Weblink (accessed on 3/10/13):
http://maps.warwickshire.gov.uk/iareports/environmental_issues/

For peer review only - http://bmjopen.bmj.com/site/about/guidelines.xhtml

Figure legends

Figure 1 : Warwickshire map with 2010 air quality index (all components of air pollution combined) displayed by ward (left; Warwickshire map with number of heart failure hospital admissions per 1000 population between 2005 – 2013 displayed by ward(centre) and Warwickshire map with total heart failure deaths per 1000 population between 2007 – 2012 displayed by ward(right).

[FIGURE 1 ABOUT HERE]

Figure 2: Left: Unadjusted total residual spatial effects of morbidity risk associated with heart failure, at ward level in Warwickshire. Shown are the posterior means. Right: Corresponding posterior probabilities at 80% nominal level.

[FIGURE 2 ABOUT HERE]

Red coloured – high risk
Green coloured – low risk

Black coloured – significant positive spatial effect
White coloured- significant negative spatial effect
Grey coloured – no significant effect

Figure 3: Left: Total residual spatial effects of morbidity risk associated with heart failure, at ward level in Warwickshire. Shown are the posterior means of the full model (IMD 2010, Over 50, and the 4 indicators of air pollution). Right: Corresponding posterior probabilities at 80% nominal level.

[FIGURE 3 ABOUT HERE]

Red coloured – high risk
Green coloured – low risk

Black coloured – significant positive spatial effect
White coloured- significant negative spatial effect
Grey coloured – no significant effect

Figure 4: Left: Unadjusted total residual spatial effects of mortality risk associated with heart failure, at ward level in Warwickshire. Shown are the posterior means. Right: Corresponding posterior probabilities at 80% nominal level.

[FIGURE 4 ABOUT HERE]

Red coloured – high risk
Green coloured – low risk

Black coloured – significant positive spatial effect
White coloured- significant negative spatial effect
Grey coloured – no significant effect

Figure 5: Left: Total residual spatial effects of mortality risk associated with heart failure, at ward level in Warwickshire. Shown are the posterior means of the full model (IMD 2010, Over 50, and the 4 indicators of air pollution). Right: Corresponding posterior probabilities at 80% nominal level.

[FIGURE 5 ABOUT HERE]

Red coloured – high risk
Green coloured – low risk

Black coloured – significant positive spatial effect
White coloured- significant negative spatial effect
Grey coloured – no significant effect

Figure 6 – Warwickshire map displaying the 2010 levels of deprivation (expressed as Multiple Deprivation Scores) by LSOAs. Produced by the Warwickshire Observatory (left) and Warwickshire map displaying the 2008 midyear estimates of % of people over the age of 50 by SOA. Produced by the Warwickshire Observatory (right).

For peer review only - http://bmjopen.bmj.com/site/about/guidelines.xhtml

Table 1: Posterior mean (PM) of fixed effects estimates of heart failure admission and mortality across air pollutions indicators (Warwickshire 2005-2013)

**Variable**	Heart failure admission PM & 95%CI‡	Heart failure mortality PM & 95%CI †
Constant	6.19 (3.84, 8.97)	4.13 (1.18, 7.43)
Nitrogen dioxide indicator (No2)	3.35 (1.89, 4.99)	4.30 (1.68, 7.37)
Sulphur dioxide indicator (So2))	7.75 (-3.84, 17.99)	11.02 (-1.21, 22.41)
Particulates indicator (Pm)	-12.93 (-20.41, -6.54)	-14.69 (-23.46, -6.50)
Benzene indicator (Ben)	[31.9:8.36, 55.85]	25.98 (-6.62, 54.22)
Over 50 years %	0.70(-0.63, 1.98)	1.60, (0.47, 2.92)
IMD 2010 score	0.02 (0.01, 0.03)	0.00 (-0.01, 0.01)

‡ Spatially adjusted posterior mean (PM) from Bayesian geo-additive regression models after controlling for fixed effect of all air pollutions indicators: Mono-nitrogen Oxide (NOx), sulphur dioxide (So2), particulate matter (Pm), benzene (Ben) and combined index and the county of residence (spatial effects). †Spatially adjusted posterior mean (PM) from Bayesian geo-additive regression models after controlling for fixed effect of all air pollutions indicators: Mono-nitrogen Oxide (NOx), sulphur dioxide (So2), particulate matter (Pm), benzene (Ben) and combined index and the county of residence (spatial effects).

For peer review only

Spatial variation of Heart Failure and Air Pollution in Warwickshire, UK: An investigation of small scale variation at the ward-level

Oscar Bennett MB BChir[1], *Ngianga-Bakwin Kandala PhD*[2, 3], *Chen Ji PhD*[2], *John Linnane MD* FFPHM[1], *Aileen Clarke MD PhD*[2]

[1] Public Health Warwickshire, Communities Group, Warwickshire County Council ,Shire Hall, Barrack Street, Warwick, CV34 4RL, UK.
[2] Division of Health Sciences, University of Warwick Medical School, Coventry, CV4 7AL, UK.
[3] Division of Epidemiology and Biostatistics, School of Public Health, University of Witwatersrand, Johannesburg , South Africa.

For peer review only

Running title: Heart Failure and Air Pollution in Warwickshire

Keywords: Warwickshire, Heart Failure, Air Pollution, epidemiology, spatial analyses

Correspondence to:

Ngianga-Bakwin Kandala, PhD
Division of Health Sciences
University of Warwick Medical School
Medical School Building
Gibbet Hill Campus
Coventry CV4 7AL
United Kingdom
Tel: + 44 (0) 2476150541
Fax:+ 44 (0) 2476528375
Email: N-B.Kandala@warwick.ac.uk

For peer review only - http://bmjopen.bmj.com/site/about/guidelines.xhtml

Abstract

Objectives: To spatially map the morbidity and mortality caused by heart failure within Warwickshire to characterise and quantify any influence of air pollution on these risks.

Design: Cross-sectional.

Setting: Warwickshire, United Kingdom

Participants: Data from all of the 105 current Warwickshire County wards were collected on hospital admissions and deaths due to heart failure.

Results

In multivariate analyses, the presence of a higher NOx in a ward [3.35:1.89, 4.99], Ben [31.9:8.36, 55.85] and IMD [0.02: 0.01, 0.03], were consistently associated with a higher risk of heart failure morbidity. Pm [-12.93:-20.41, -6.54] was negatively associated with the risk of heart failure morbidity. No association was found between So2 and heart failure morbidity. The risk of heart failure mortality was higher in wards with a higher NOx [4.30:1.68, 7.37] and wards with more inhabitants 50+ years old [1.60: 0.47, 2.92]. Pm was negatively associated [-14.69: -23.46, -6.50] with heart failure mortality. So2, Ben, and IMD score were not associated with heart failure mortality.

There was a prominent variation in heart failure morbidity and mortality risk across wards, the highest risk being in the regions around Nuneaton and Bedworth.

Conclusion

This study showed distinct spatial patterns in heart failure morbidity and mortality, suggesting the potential role of environmental factors beyond individual-level risk factors. Air pollution levels should therefore be taken into account when considering the wider determinants of public health and the impact that changes in air pollution might have on the health of a population.

For peer review only - http://bmjopen.bmj.com/site/about/guidelines.xhtml

ARTICLE FOCUS

- Air pollution has been linked to the development and exacerbation of a number of health problems including cardiovascular diseases such as heart failure.
- Heart failure is a serious condition that unfortunately affects many people in the UK. This link seems especially clear for cardiovascular and respiratory diseases such as ischaemic heart disease, heart failure, asthma, influenza, and lung cancer.
- It is well established that the ambient levels of air pollution in a region can have an impact on the health status of the population which inhabits it

KEY MESSAGES

1. Using a novel approach, this study has characterised and quantified the potential role of environmental factors (air pollutants) at the county-level.
2. The presence of a higher Mono-nitrogen Oxide (NOx) in a ward, benzene and poor Index of Multiple Deprivation (IMD) score were consistently associated with a higher risk of heart failure morbidity. Particulate matter (Pm) was negatively associated with the risk of heart failure morbidity.
3. The risk of heart failure mortality was higher in wards with a higher NOx and wards with more inhabitants 50+ years old. Pm was negatively associated with heart failure mortality.
4. Air pollution overall, when all pollution components were brought together into a combined index, was positively associated with both heart failure morbidity and mortality across Warwickshire.
5. There was a striking variation in heart failure morbidity and mortality risk across wards, the highest risk being in the regions around Nuneaton and Bedworth.

STRENGTHS AND LIMITATIONS

- The model employed a fully Bayesian approach using Markov Chain Monte Carlo (MCMC) techniques for inference and model checking [11, 12]

- A single air pollution measurement in 2010 was used and it was assumed that there was no significant change in this value over the 2005-2013 periods that mortality and hospital admission data was gathered from.
- The cross-sectional nature of the study unfortunately does not allow us to establish temporality and thus clearly demonstrate causality of the observed associations.
- This was an ecological study, analysing characteristics and risk at the ward rather than individual level. Consequently associations and conclusions found from the aggregate data may not be directly applicable to individuals.

For peer review only - http://bmjopen.bmj.com/site/about/guidelines.xhtml

Introduction

Air pollution has been linked to the development and exacerbation of a number of health problems. This link seems especially clear for cardiovascular and respiratory diseases such as ischaemic heart disease, heart failure, asthma, influenza, and lung cancer [(1, 2, 3, 4)]. It is well established that the ambient levels of air pollution in a region can have an impact on the health status of the population which inhabits it [(5)]. Air pollution levels should therefore be taken into account when considering the wider determinants of public health and the impact that changes in air pollution might have on the health of a population.

Warwickshire is an English county within the United Kingdom approximately 70 miles North-West of London. The issue of air pollution has been highlighted in Warwickshire recently by the setting up of Air Quality Management Areas (AQMAs) in a number of different parts of the county. These are specific areas in the county that have been identified as places where, without a focused local council strategy to reduce air pollution, future government targets for air pollutant concentrations may not be met. Within Warwickshire the specific air pollutant that has been identified as a problem and led to these special areas being setup is mono-nitrogen oxides (NOx)[(6)].

The objective of the present study was therefore to look for any relationship between levels of air pollution and the morbidity and mortality associated with heart failure within Warwickshire in the last few years (2005-2013 for morbidity, 2007-2012 for mortality). We examined a range of traditional air pollutants for an ecological association with heart failure morbidity and mortality at the county level. Furthermore, the present analysis attempted to highlight spatial patterns in heart failure morbidity and mortality risk within the county, after multiple adjustments for proximate, county-level factors. The geographic locations of wards within the county can be considered as proxy measures of many other unmeasured factors such as availability and access to health services, individual health seeking behaviour,

For peer review only - http://bmjopen.bmj.com/site/about/guidelines.xhtml

preventive ward policy and general ward factors. Such estimates might illustrate how much can be learned by detailed exploratory analyses as well as how these data can be used to strategically inform policy aiming at the prevention and management of serious conditions such as heart failure in these settings.

The evidence from published literature for the link between air pollution and health problems

Studies have shown an effect on the health of populations caused by both long term exposure as well as short term "spikes" in local air pollution levels[5].

A relevant systematic review and meta-analysis has been published recently in the Lancet [2]. It pooled the results of studies that have been conducted worldwide looking at the temporal relationship between the levels of a number of different air pollutants with local heart failure hospital admission rates and mortality rates. This showed a clear link between short term rises in all types of air pollution (except ozone) and rises in hospital admissions and mortality due to heart failure. This study did not look at any potential effects from long term exposure to air pollution. However it did provide strong evidence that air pollution can 'exacerbate' heart failure, increasing the likelihood that a patient with existing heart failure will become sufficiently unwell to require hospital admission, or even die.

The ESCAPE study[3], published recently in the Lancet Oncology, pooling the results of 17 cohort studies from around Europe looked at the ambient levels of air pollution in an area (particulate matter and NOx) and the incidence of lung cancers in the inhabitants of this area during many years of follow up (mean 12.8 years). This was intended to look at the risk associated with long term exposure to air pollution. A statistically significant correlation was found between the levels of particulate matter air pollution and the local incidence of lung cancer diagnoses. Particulate matter with a diameter of less than 10 μm had a hazard ratio of 1.22 [95% CI 1.03-1.45] per (10 μg/cubic meter). In other words, for every increase in

For peer review only - http://bmjopen.bmj.com/site/about/guidelines.xhtml

particulate matter pollution of 10 μg/cubic meter there was a corresponding immediate increase in the chance of being diagnosed with lung cancer of 22% (95% CI 3% - 45%). There was also a correlation between traffic volume within 100m of a residence and a modest increase in the rate of lung cancer (Hazard ratio 1.09 (CI 0.99-1.21)). No similar correlation was found with NOx air pollution. This suggests that long term exposure to higher levels of particulate matter air pollution may increase the incidence of lung cancer in a population. Another study published in the United States in 2004 looked at the long term effect of exposure to particulate matter air pollution and the mortality attributed to different cardiovascular and respiratory diseases in different areas of the United States[(4)]. A good correlation was found between the degree of long term exposure to particulate matter air pollution and increases in mortality from cardiovascular diseases, including heart failure. Interestingly this was not found to be the case for most respiratory diseases. There was also an element of the study that looked at the effect of a person's smoking status on the mortality statistics. This found, as expected, a strong link between mortality and smoking. However it also found that air pollution contributed additional cardiovascular mortality risk on top of that attributed to smoking. This was at least an additive, if not a synergistic effect.

Methods

Study data

In order to carry out this project, data was collected about the geographical distribution of air pollution within Warwickshire. This data included information about each of four individual components of air pollution (NOx, sulphur dioxide, particulate matter, and benzene) which could then be united into a combined index (all of the contributions added together). A single recorded level from 2010 of each air pollutant for each ward was used in the study. This was then compared to collected data about:

For peer review only - http://bmjopen.bmj.com/site/about/guidelines.xhtml

For peer review only

(i) The geographical distribution of home addresses of patients who were admitted to hospital because of heart failure or a complication of heart failure. Hospital admission rate in an area due to heart failure was used as a proxy indicator for the level of heart failure morbidity within that area.

(ii) The geographical distribution of home addresses of patients who died from heart failure, or whose death was contributed to by heart failure.

These data were collected by the Warwickshire Observatory, which is part of the Warwickshire County Council in charge of collecting and handling statistics relating to the county. Mortality data for the analysis was supplied via the Warwickshire Public Health Intelligence Team and was sourced from the Public Health Mortality Files, Office for National Statistics. Hospital admissions data was accessed via the Ventris Business Intelligence System, Arden Commissioning Support Unit."

Ward level population data were obtained from the 2011 census. Warwickshire is divided into 105 wards. Data from all of the 105 current Warwickshire County wards were collected. The potential confounding variables of firstly age structure of a population within a ward and secondly levels of social deprivation within a ward were identified [7,8]. These were then controlled for in the second stage of the statistical analysis (see below).

Information about the age structure of wards was obtained from the Office of National Statistics mid 2010 estimates (obtained from the Warwickshire Observatory website)[9]. The representative statistical value of "percentage (%) of population above age 50" was used as an indicator of wards with a higher proportion of older people.

Information about social deprivation was obtained from the English Indices of Deprivation published by the Department for Communities and Local Government [10]. The Index of Multiple Deprivation (IMD) averaged across each ward was used as an indicator of the level of deprivation within the wards of Warwickshire.

For peer review only - http://bmjopen.bmj.com/site/about/guidelines.xhtml

The information on crude observed air pollution level distribution, heart failure hospital admission rates and mortality rates were then represented on maps of Warwickshire (**Figure 1**).

Statistical Analysis

To account for spatial autocorrelation in observed heart failure hospital admission and mortality rates at the ward level, in Warwickshire, we applied a unified approach to account for possible air pollution effects of environmental risk factors. This was achieved using a geo-additive semi-parametric mixed model. The model employed a fully Bayesian approach using Markov Chain Monte Carlo (MCMC) techniques for inference and model checking [11, 12]. Response variables were defined as the count (per 1000 population) of heart failure morbidity or mortality in a ward (Poisson model): $y_i / \eta_i, \delta \sim B(\eta_i, \delta)$ for a binomial formulation.

(Poisson model): $y_i / \eta_i, \delta \sim B(\eta_i, \delta)$ (1) for a binomial formulation and a geo-additive semi-parametric predictor $\mu_i = h(\eta_i)$:

$$\eta_i = f1(xi1) + \ldots + fp(xip) + fspat(s_i) + \varepsilon_i \qquad (2)$$

where h is a known response function with a poison link function, $f_1, \ldots, f_p$ are non-linear smoothed effects of the metrical covariates (time in years), and $f_{spat}(s_i)$ is the effect of the spatial covariate $s_i \in \{1, \ldots, S\}$ labelling the ward in Warwickshire. Regression models with predictors such as those in equation 2 are sometimes referred to as geo-additive models. P-spline priors were assigned to the functions $f1, \ldots, f_p$, and a Markov random field prior was used for $f_{spat}(s_i)$. More detailed information about the modelling approach can be found elsewhere [11, 13]. The standard measure of effect was the posterior mean (PM) and 95% credible region (CR).

The analysis was carried out using version 2.0.1 of the BayesX software package, which permits Bayesian inference based on MCMC simulation techniques[13]. Multivariate Bayesian geo-additive regression models were used to evaluate the significance of the posterior mean

For peer review only - http://bmjopen.bmj.com/site/about/guidelines.xhtml

(PM) determined for the fixed effects and spatial effects between air pollution and the morbidity and mortality from heart failure within Warwickshire. Each component of air pollution was looked at separately and then also the combined index in unadjusted models. Next, fully adjusted multivariate Bayesian geo-additive regressions analysis were performed to look again for statistically significant correlation between these variables, but this time further controlling for any influence from age structure or social deprivation.

Results

Figure 1 and **Figure 6** display the observed data collected for this study on maps using graduated colouring to represent data value categories within each ward.

Observed Air Quality Map

Figure 1left was produced using 2010 data from the Warwickshire Observatory. It is based on a combined air quality indicator which is a combination of information about the contribution to air pollution from NOx, sulphur dioxide, particulate matter, and benzene. The Warwickshire Observatory description of this index reads as follows:

"Combined Air Quality Indicator (estimates of emissions for four pollutants: benzene, nitrogen dioxide, sulphur dioxide and particulates) for small areas (modelled to 1 km grid squares) where an index value of 1 is equivalent to the national standard for each pollutant. The values are then summed so an overall score of 4 would represent all four pollutants being present at the national standard level."**[14]** These specific standards are described in the Air Quality Standards Regulations 2010.

The geographical pattern of the observed air pollution across the county shows a higher level of air pollution near the more urban centres of Birmingham and Coventry. The proximity of parts of the county to motorways such as the M6 and M42 could also be a contributing factor

For peer review only - http://bmjopen.bmj.com/site/about/guidelines.xhtml

to the observed pattern. There was no place in the county where the level of any pollutant exceeded the national standard in 2010.

Heart Failure Hospital Admission Map

Figure 1centre shows the geographical distribution of the density of home addresses of patients admitted to hospital with a diagnosis of heart failure (or exacerbation of heart failure) within the April 2005- April 2013 period.

Heart Failure Mortality Map

Figure 1right shows the geographical distribution of the density of home addresses of patients who died either directly or in part from heart failure in the 2007-2012 (inclusive) periods.

Table 1 (left panel) displays posterior means of heart failure admission across the selected covariates following multivariate Bayesian geo-additive regression analyses.

The Posterior mean (PM) & 95% Credible Region (CR) of overall hospital admission rates due to heart failure and mortality rates from heart failure were [6.19 (3.84, 8.97)] and [4.13 (1.18, 7.43)] (Table 2) respectively. On average, the presence of a higher Mono-nitrogen oxide indicator (NOx) in a ward [Posterior mean (PM) & 95% Credible Region (CR): 3.35 (1.89, 4.99)], Benzene indicator (Ben) [31.9 (8.36, 55.85)] and IMD 2010 score [0.02 (0.01, 0.03)], were consistently associated with higher risk of heart failure morbidity. The particulates indicator (Pm) [-12.93 (-20.41, -6.54)] was negatively associated with the risk of heart failure morbidity. Sulphur dioxide indicator (So2) was not associated with heart failure morbidity.

For peer review only - http://bmjopen.bmj.com/site/about/guidelines.xhtml

Table 1 (right panel) shows the same corresponding results for heart failure mortality. This table shows that the risk of heart failure mortality was higher in wards with a higher Mono-nitrogen oxide indicator (NOx) [4.30 (1.68, 7.37)] and wards with more inhabitants over 50 years old [1.60 (0.47, 2.92)]. The particulates indicator (Pm) was negatively associated with heart failure mortality. The sulphur dioxide indicator (So2), Benzene indicator (Ben), and IMD score were not associated with heart failure mortality.

For peer review only

The combined air pollution index (all indicators averaged together) when incorporated into a separate model combining age and social deprivation was significantly positively associated with both heart failure morbidity [1.39 (0.87, 1.81)] and mortality [1.79 (0.85, 2.55)] across the county.

In **Figure 2** and **Figure 4**, the left-hand maps show the unadjusted estimates of posterior total residual ward means of heart failure morbidity and mortality respectively. In **Figure 3** and **Figure 5**, the left-hand maps show the adjusted PM after multiple adjustment for the geographical location, taking into account the auto-correlation structure in the data, the uncertainty in the ward level and all ward-level risk factors (air pollution components, age, social deprivation) for heart failure morbidity and mortality respectively. The red colour indicates the maximum posterior mean recorded while green denotes the lowest mean. The right-hand maps in **Figures 2**, **3**, **4** and **5** show the 95% posterior probability of heart failure and mortality, which indicate the statistical significance associated with the total excess risk. Black colour indicates a negative spatial effect (associated with increased risk of heart failure and mortality), white colour a positive effect (a decreased risk) and grey colour a non-significant effect.

In general, there was consistently higher heart failure morbidity risk in northern wards particularly around Nuneaton and Bedworth and lower heart failure morbidity risks in the

For peer review only - http://bmjopen.bmj.com/site/about/guidelines.xhtml

southern wards particularly within the district of Stratford-on-Avon. However, all this variation could partially be accounted for within the model generated within this study (taking into account air pollution, age, and social deprivation levels).

Heart failure mortality risk was, in general, higher in northern wards around Nuneaton and Bedworth as well as in some central areas around Warwick, Royal Leamington Spa, and Kenilworth. Heart failure mortality risk was again lower in more southern wards particularly within Stratford-on-Avon. Unlike with morbidity however, our model could not explain all this variation in heart failure mortality. Even after taking into account air pollution, age, and social deprivation the rate of death from heart failure remained significantly higher than would be expected in areas within and around Nuneaton, Bedworth, Warwick, Royal Leamington Spa, and Kenilworth.

In sensitivity analyses, we tested several models and our results were not substantially altered after removing one or two pollutant (data not shown).

Discussion

Before even considering air pollution, this study helps to demonstrate the inequality of risk from heart failure disease and death that exists for individuals living in different parts of the county of Warwickshire. There is a significant excess risk of both disease and death in more northern wards within and surrounding Nuneaton and Bedworth. Much (but not all) of this variation could be attributed to the air pollution, age structure and social deprivation that exists in these areas according to our model. Measures that seek to address air pollution and social deprivation could be expected therefore to help mitigate against cardiovascular risks within these local populations.

For peer review only - http://bmjopen.bmj.com/site/about/guidelines.xhtml

The present study corroborates the notion that air pollution is an increasingly important public health issue in Warwickshire. Higher levels of the average air pollution index looked at in this study correlated significantly with increased levels of both heart failure morbidity and mortality across the county, even after removing the effects of age structure and social deprivation. The individual component of NOx air pollution seems particularly to contribute risk to both the morbidity and mortality of heart failure within the county, suggesting that it may have a particularly detrimental effect on heart failure patients. This reinforces the importance of the AQMAs set up within the county in response to high NOx levels. Road traffic is a large contributor to air pollution within Warwickshire. Diesel engines are responsible for a large part of the NOx component of these emissions.

An unexpected result also appeared within our analysis. Particulate matter air pollution became significantly, negatively correlated with both heart failure morbidity and mortality when incorporated into our model with all risk factors taken into account and controlled for in this study. This would imply some sort of unexpected "protective influence" from particulate matter air pollution on heart failure patients. This clearly contradicts our expectations and is at odds with a wealth of existing evidence that indicates that particulate matter air pollution contributes risk to and exacerbates cardiovascular disease[4].

We offer a possible explanation for this based on the following four observations:

1. The above-mentioned negative correlation of particulate matter air pollution with heart failure morbidity and mortality in our model.
2. Particulate matter air pollution actually varied very little across the county compared to the other types of air pollution. All types of air pollution tended to decrease in rural areas, but particulate matter tended to decrease much *less* compared to the other components of air pollution. Consequently in rural areas of the county where most

For peer review only - http://bmjopen.bmj.com/site/about/guidelines.xhtml

types of air pollution are significantly lower, particulate matter pollution was relatively higher compared to NOx, Benzene, and SO2.

3. There seems to be a high risk of heart failure deaths in urban centres (particularly Nuneaton, Bedworth, Warwick, Royal Leamington Spa, and Kenilworth), higher than can be explained by our model.
4. Conversely, there seems to be a particularly low risk of heart failure deaths in some rural areas within the western part of Stratford-on-Avon, lower than can be explained by our model.

A possible hypothesis based on these observations is that there is an additional factor influencing the morbidity and mortality of heart failure not looked at in this study, namely the urban/rural nature of a patient's living environment. It could be the case that living in an urban environment contributed risk and living in a rural area provided protection against heart failure morbidity and mortality. This would be an effect in *addition* to any increase in air pollution or social deprivation within urban settings compared to rural settings. This could certainly in principle be plausible, with people in rural areas perhaps doing more physical activity, eating more healthily etc. If this were the case it would explain the excess deaths in urban centres found in this study. It could also be responsible for the unexpected protective factor attributed to particulate matter air pollution in our analysis. Given that the particulate matter component of air pollution is *relatively* higher than the other components in rural areas, the protection that living in rural areas affords individuals could be misleadingly attributed (in our statistical analysis) to the particulate matter component of air pollution present in these areas. Further work will need to be done looking into this possible link between urban/rural living environments and heart failure morbidity and mortality. It could be very revealing to carefully characterise this effect if it indeed exists, as it may be an indication of unrecognised cardiovascular risk/protective factors associated with urban/rural living that exists within Warwickshire.

For peer review only - http://bmjopen.bmj.com/site/about/guidelines.xhtml

However, it is also important to bear in mind that this is an ecological study and all the relationships picked up between variables in this study have been found using aggregate data at the ward level (number over 50 years of age, average IMD score, average air pollution across ward, overall numbers of deaths and hospital admissions due to heart failure). It is not always a trivial task to extrapolate the conclusions drawn from such a study down to the level of individuals. Such a task would involve drilling down to individual level data and repeating the analysis, a task that was beyond the scope of this particular study. It is certainly possible that the unexpected negative correlation between particulate matter air pollution and heart failure could disappear when data is analysed at the individual level – an example of an ecological fallacy. Consequently it would be prudent to regard the results from the individual components of air pollution with cautious interest, rather than viewing them as proof of any real effects.

However, despite these caveats, this study has been able to provide some helpful information at the population level worthy of consideration. A health inequality has been revealed, and the manner with which this inequality is influenced by age, social deprivation, and the combined index of air pollution has been demonstrated. Such information should help inform policy decisions that would influence society at a population level and hopefully improve public health in the long run.

There are some limitations in this study worth considering that result from assumptions made along the way. A single air pollution measurement in 2010 was used and it was assumed that there was no significant change in this value over the 2005-2013 periods that mortality and hospital admission data was gathered from. The resulting cross-sectional nature of the study does not allow establishing temporality and thus causality of the observed associations. There was also no way to determine the length of time that individual members of the population within a ward had lived in that area, and thus how long they had been exposed to the

For peer review only - http://bmjopen.bmj.com/site/about/guidelines.xhtml

measured ambient air pollution level. It was assumed that people with home addresses in a ward were exposed significantly to the levels of air pollution in that ward. Finally, as already mentioned, this was an ecological study, using aggregate data of risk factors to look for associations with aggregate data of morbidity and mortality. It is not always possible to apply such associations from a population level down to the individuals within that population.

In summary, this study has provided a number of interesting results. Firstly it has helped to quantify and map the inequality that exists across different parts of Warwickshire with regards to heart failure risk. It has also provided some interesting circumstantial evidence of a link between heart failure morbidity and air pollution. Finally it has also given a suggestion of a possible link between living in urban environments and a higher risk of cardiovascular disease, and a corresponding lower risk from living in rural environments. More work will need to be done to look into this particular possibility. It would be informative to run this type of analysis whilst factoring in the influence of a person's distance from their nearest urban centre. This urban/rural factor should be further explored and mined for additional information as it could be an indication of hitherto unconsidered factors influencing the health status of the population of Warwickshire and possibly further afield.

In order to determine the validity of our conclusions at the individual level further work would need to be done analysing the available data from individual patients (risks and outcomes). Such work could help to characterise the true effect of different components of air pollution at the individual level. It would also be interesting to determine if the different components of air pollution act as effect modifiers on each other.

It would be possible to look at the effects of air pollution variation in the shorter term as well. For example, looking at how local "spikes" in air pollution affect the rates of hospital admissions locally immediately following it. This could be done in Leamington Spa where there is an air quality monitoring station constantly measuring the levels of air pollutants.

For peer review only - http://bmjopen.bmj.com/site/about/guidelines.xhtml

Other health problems, such as ischaemic heart disease and respiratory diseases, have been linked with air pollution as well and it could be informative to also look into these links locally.

Contributorship statement:

OB conceived the idea, analyzed the data, contributed to formulating the results and wrote the first draft. N-B K analyzed the data, advised on statistical aspects, contributed to formulating the results, wrote the second draft. CJ analyzed the data. JL helped coordinate the project and co-wrote the final draft; AC coordinated the project, advised on all aspects and co-wrote the final draft.

Funding:

This paper presents independent research supported by the National Institute for Health Research (NIHR) Collaborations for Leadership in Applied Health Research and Care West Midlands. The views expressed are those of the author(s) and not necessarily those of the NHS, the NIHR or the Department of Health.

Competing interests: The authors declared no conflict of interest.

Data sharing: Not application

References

1. Particulate Air Pollution and Hospital Emergency Room Visits for Asthma in Seattle, Joel Schwartz, Daniel Slater, Timothy V. Larson, William E. Pierson, and Jane Q. Koenig. American Review of Respiratory Disease, Vol. 147, No. 4 (1993), pp. 826-831.
2. Global association of air pollution and heart failure: a systematic review and meta-analysis, Anoop SV Shah, Jeremy P Langrish,Harish Nair,David A McAllister, Amanda L Hunter, Prof Ken Donaldson, Prof David E Newby, Dr Nicholas L Mills. The Lancet, Early Online Publication, 10 July 2013 DOI: 10.1016/S0140-6736(13)60898-3
3. Air pollution and lung cancer incidence in 17 European cohorts: prospective analyses from the European Study of Cohorts for Air Pollution Effects (ESCAPE), Raaschou-Nielsen O, Andersen ZJ, et al. Lancet Oncol. 2013 Aug;14(9):813-22
4. Cardiovascular Mortality and Long-Term Exposure to Particulate Air Pollution, Epidemiological Evidence of General Pathophysiological Pathways of Disease C. Arden Pope III, Richard T. Burnett, et al. Circulation. 2004; 109: 71-77

For peer review only - http://bmjopen.bmj.com/site/about/guidelines.xhtml

5. Air pollution and health, Bert Brunekreef, Stephen T Holgate, The Lancet, Volume 360, Issue 9341, 19 October 2002, Pages 1233–1242
6. Department of Environment, Food and Rural Affairs (DEFRA), department website, weblink (accessed 2/12/13): http://aqma.defra.gov.uk/aqma/local_authorities.php?la_id=296
7. Epidemiology, pathophysiology, and etiology of congestive heart failure in older adults. Rich MW. J Am Geriatr Soc. 1997 Aug;45(8):968-74.
8. Influence of socioeconomic deprivation on the primary care burden and treatment of patients with a diagnosis of heart failure in general practice in Scotland: population based study. F A McAlister, N F Murphy, et al. BMJ. 2004 May 8; 328(7448): 1110.
9. Warwickshire Observatory website – ONS population estimates. Web link (accessed on 3/10/13): http://www.warwickshireobservatory.org/observatory/observatorywcc.nsf/05a17cfbd3bcf85d802572920033cf0f/bfb8b0a1d34f274c802572c00048108f?OpenDocument
10. Deprivation data produced by the Department for Communities and Local Government. File located at web link (accessed on 3/10/13): https://www.gov.uk/government/publications/english-indices-of-deprivation-2010 Ward based population weighted averages calculated from LSOA specific data by the London Health Observatory and North East Public Health Observatory.
11. Fahrmeir L, Lang S. Bayesian Inference for Generalized Additive Mixed Models Based on Markov Random Field Priors. *Applied Statistics (JRSS C)*. 2001;50:201-20.
12. Kandala N-B, Tigbe W, Manda S, Stranges S. Spatial Variation of Predictors of Hypertension in Sub-Saharan Africa: A Case Study of South-Africa. American Journal of Hypertension 2013. 10.1093/ajh/hps063.
13. Belitz C, Brezger A, Kneib T, Lang S. BayesX Software for Bayesian Inference in Structured Additive Regression Models Version 2.0.1, 2012. Accessed at: http://www.stat.uni-muenchen.de/~bayesx/manual/methodology_manual.pdf
14. Taken from the Warwickshire Observatory Quality of Life in Warwickshire 2012/13 report. Weblink (accessed on 3/10/13): http://maps.warwickshire.gov.uk/iareports/environmental_issues/

Author's contributions

OB conceived the idea, analyzed the data, contributed to formulating the results and wrote the first draft.

N-B K analyzed the data, advised on statistical aspects, contributed to formulating the results, wrote the second draft. CJ analyzed the data. JL helped coordinate the project and co-wrote the final draft; AC coordinated the project, advised on all aspects and co-wrote the final draft.

For peer review only - http://bmjopen.bmj.com/site/about/guidelines.xhtml

Figure legends

Figure 1 : Warwickshire map with 2010 air quality index (all components of air pollution combined) displayed by ward (left; Warwickshire map with number of heart failure hospital admissions per 1000 population between 2005 – 2013 displayed by ward(centre) and Warwickshire map with total heart failure deaths per 1000 population between 2007 – 2012 displayed by ward(right).

[FIGURE 1 ABOUT HERE]

Figure 2: Left: Unadjusted total residual spatial effects of morbidity risk associated with heart failure, at ward level in Warwickshire. Shown are the posterior means. Right: Corresponding posterior probabilities at 80% nominal level.

[FIGURE 2 ABOUT HERE]

Red coloured – high risk
Green coloured – low risk

Black coloured – significant positive spatial effect
White coloured- significant negative spatial effect
Grey coloured – no significant effect

Figure 3: Left: Total residual spatial effects of morbidity risk associated with heart failure, at ward level in Warwickshire. Shown are the posterior means of the full model (IMD 2010, Over 50, and the 4 indicators of air pollution). Right: Corresponding posterior probabilities at 80% nominal level.

[FIGURE 3 ABOUT HERE]

Red coloured – high risk
Green coloured – low risk

Black coloured – significant positive spatial effect
White coloured- significant negative spatial effect
Grey coloured – no significant effect

Figure 4: Left: Unadjusted total residual spatial effects of mortality risk associated with heart failure, at ward level in Warwickshire. Shown are the posterior means. Right: Corresponding posterior probabilities at 80% nominal level.

[FIGURE 4 ABOUT HERE]

Red coloured – high risk
Green coloured – low risk

Black coloured – significant positive spatial effect
White coloured- significant negative spatial effect
Grey coloured – no significant effect

Figure 5: Left: Total residual spatial effects of mortality risk associated with heart failure, at ward level in Warwickshire. Shown are the posterior means of the full model (IMD 2010, Over 50, and the 4 indicators of air pollution). Right: Corresponding posterior probabilities at 80% nominal level.

[FIGURE 5 ABOUT HERE]

Red coloured – high risk
Green coloured – low risk

Black coloured – significant positive spatial effect
White coloured- significant negative spatial effect
Grey coloured – no significant effect

Figure 6 – Warwickshire map displaying the 2010 levels of deprivation (expressed as Multiple Deprivation Scores) by LSOAs. Produced by the Warwickshire Observatory (left) and Warwickshire map displaying the 2008 midyear estimates of % of people over the age of 50 by SOA. Produced by the Warwickshire Observatory (right).

For peer review only - http://bmjopen.bmj.com/site/about/guidelines.xhtml

For peer review only

Table 1: Posterior mean (PM) of fixed effects estimates of heart failure admission and mortality across air pollutions indicators (Warwickshire 2005-2013)

**Variable**	Heart failure admission PM & 95%CI‡	Heart failure mortality PM & 95%CI †
Constant	6.19 (3.84, 8.97)	4.13 (1.18, 7.43)
Nitrogen dioxide indicator (No2)	3.35 (1.89, 4.99)	4.30 (1.68, 7.37)
Sulphur dioxide indicator (So2))	7.75 (-3.84, 17.99)	11.02 (-1.21, 22.41)
Particulates indicator (Pm)	-12.93 (-20.41, -6.54)	-14.69 (-23.46, -6.50)
Benzene indicator (Ben)	[31.9:8.36, 55.85]	25.98 (-6.62, 54.22)
Over 50 years %	0.70(-0.63, 1.98)	1.60, (0.47, 2.92)
IMD 2010 score	0.02 (0.01, 0.03)	0.00 (-0.01, 0.01)

‡ Spatially adjusted posterior mean (PM) from Bayesian geo-additive regression models after controlling for fixed effect of all air pollutions indicators: Mono-nitrogen Oxide (NOx), sulphur dioxide (So2), particulate matter (Pm), benzene (Ben) and combined index and the county of residence (spatial effects). †Spatially adjusted posterior mean (PM) from Bayesian geo-additive regression models after controlling for fixed effect of all air pollutions indicators: Mono-nitrogen Oxide (NOx), sulphur dioxide (So2), particulate matter (Pm), benzene (Ben) and combined index and the county of residence (spatial effects).

For peer review only

For peer review only - http://bmjopen.bmj.com/site/about/guidelines.xhtml

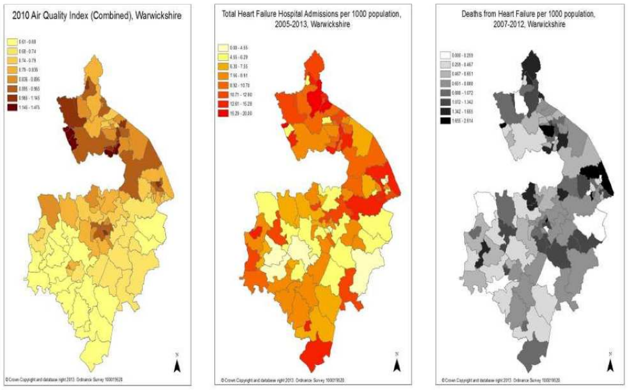

Figure 1
297x420mm (300 x 300 DPI)

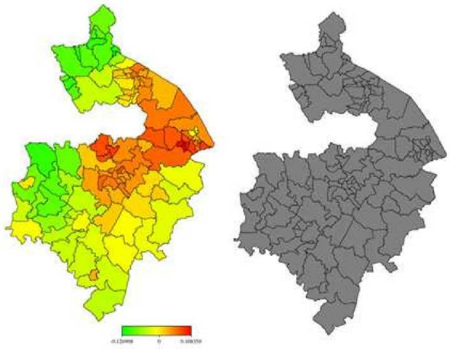

Figure 2
297x420mm (300 x 300 DPI)

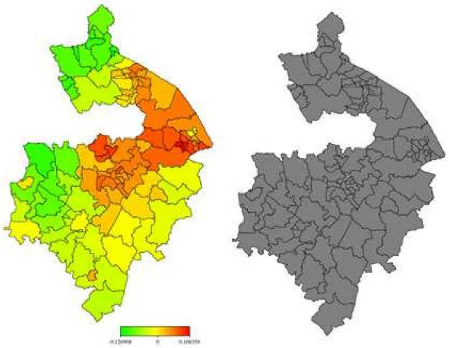

Figure 3
297x420mm (300 x 300 DPI)

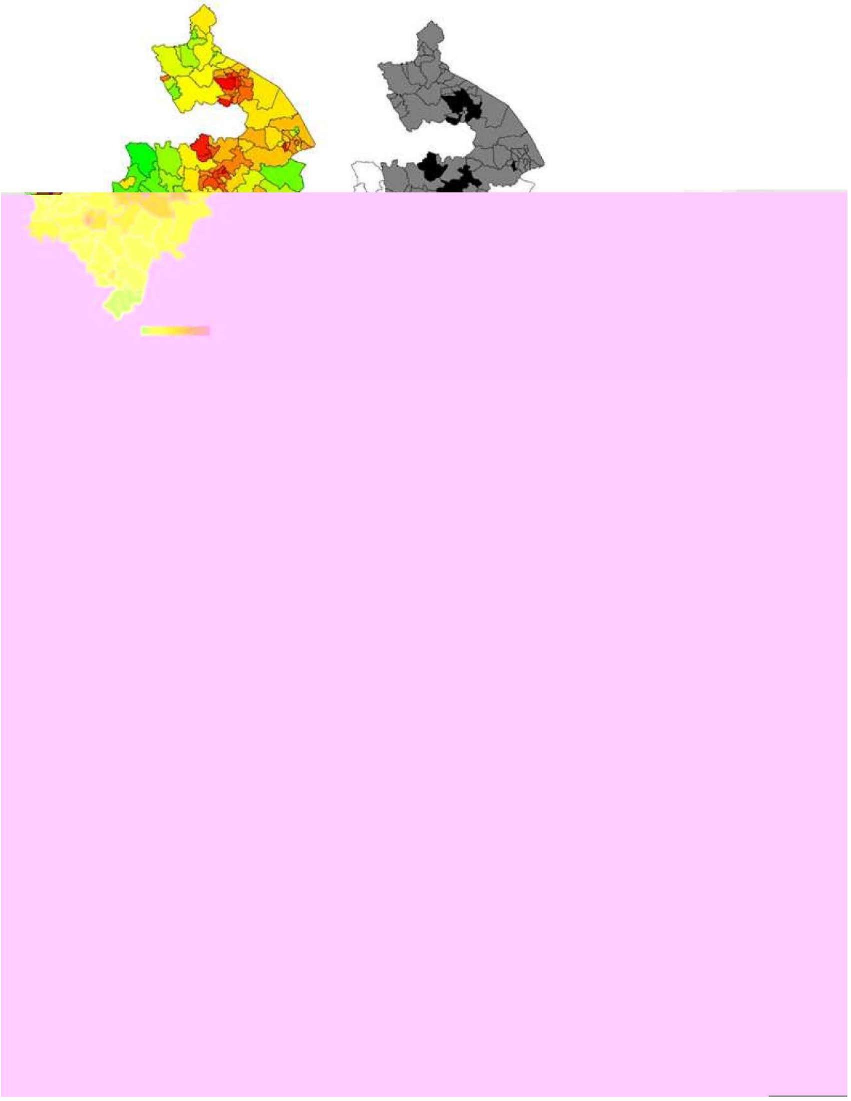

Figure 4
297x420mm (300 x 300 DPI)

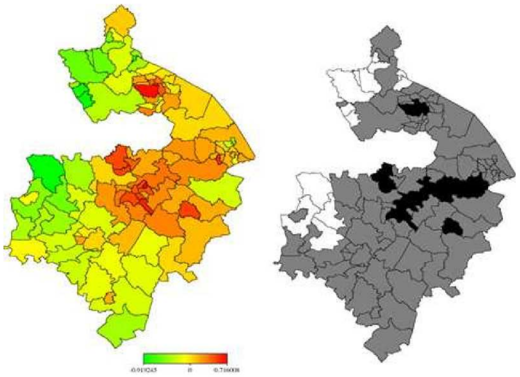

Figure 5
297x420mm (300 x 300 DPI)

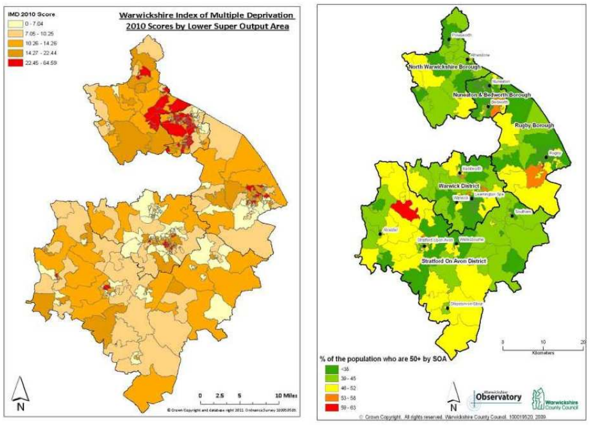

Figure 6
297x420mm (300 x 300 DPI)

STROBE 2007 (v4) Statement—Checklist of items that should be included in reports of *cross-sectional studies*

Section/Topic	Item #	Recommendation	Reported on page #
**Title and abstract**	1	(*a*) Indicate the study's design with a commonly used term in the title or the abstract	2
		(*b*) Provide in the abstract an informative and balanced summary of what was done and what was found	2
**Introduction**			
Background/rationale	2	Explain the scientific background and rationale for the investigation being reported	4
Objectives	3	State specific objectives, including any prespecified hypotheses	4,5
**Methods**			
Study design	4	Present key elements of study design early in the paper	4, 5
Setting	5	Describe the setting, locations, and relevant dates, including periods of recruitment, exposure, follow-up, and data collection	5,6
Participants	6	(*a*) Give the eligibility criteria, and the sources and methods of selection of participants	5,6,
Variables	7	Clearly define all outcomes, exposures, predictors, potential confounders, and effect modifiers. Give diagnostic criteria, if applicable	5-7
Data sources/ measurement	8*	For each variable of interest, give sources of data and details of methods of assessment (measurement). Describe comparability of assessment methods if there is more than one group	5-6
Bias	9	Describe any efforts to address potential sources of bias	*7-8,12*
Study size	10	Explain how the study size was arrived at	5
Quantitative variables	11	Explain how quantitative variables were handled in the analyses. If applicable, describe which groupings were chosen and why	7-8
Statistical methods	12	(*a*) Describe all statistical methods, including those used to control for confounding	7,8
		(*b*) Describe any methods used to examine subgroups and interactions	NA
		(*c*) Explain how missing data were addressed	NA
		(*d*) If applicable, describe analytical methods taking account of sampling strategy	NA
		(*e*) Describe any sensitivity analyses	NA
**Results**			

For peer review only

Participants	13*	(a) Report numbers of individuals at each stage of study—eg numbers potentially eligible, examined for eligibility, confirmed eligible, included in the study, completing follow-up, and analysed	2, 5,6
		(b) Give reasons for non-participation at each stage	NA
		(c) Consider use of a flow diagram	NA
Descriptive data	14*	(a) Give characteristics of study participants (eg demographic, clinical, social) and information on exposures and potential confounders	6
		(b) Indicate number of participants with missing data for each variable of interest	NA
Outcome data	15*	Report numbers of outcome events or summary measures	10-11
Main results	16	(*a*) Give unadjusted estimates and, if applicable, confounder-adjusted estimates and their precision (eg, 95% confidence interval). Make clear which confounders were adjusted for and why they were included	8
		(*b*) Report category boundaries when continuous variables were categorized	NA
		(*c*) If relevant, consider translating estimates of relative risk into absolute risk for a meaningful time period	NA
Other analyses	17	Report other analyses done—eg analyses of subgroups and interactions, and sensitivity analyses	NA
**Discussion**			
Key results	18	Summarise key results with reference to study objectives	8-10
Limitations	19	Discuss limitations of the study, taking into account sources of potential bias or imprecision. Discuss both direction and magnitude of any potential bias	11, 15
Interpretation	20	Give a cautious overall interpretation of results considering objectives, limitations, multiplicity of analyses, results from similar studies, and other relevant evidence	11-15
Generalisability	21	Discuss the generalisability (external validity) of the study results	11-15
**Other information**			
Funding	22	Give the source of funding and the role of the funders for the present study and, if applicable, for the original study on which the present article is based	NA

*Give information separately for cases and controls in case-control studies and, if applicable, for exposed and unexposed groups in cohort and cross-sectional studies.

Note: An Explanation and Elaboration article discusses each checklist item and gives methodological background and published examples of transparent reporting. The STROBE checklist is best used in conjunction with this article (freely available on the Web sites of PLoS Medicine at http://www.plosmedicine.org/, Annals of Internal Medicine at http://www.annals.org/, and Epidemiology at http://www.epidem.com/). Information on the STROBE Initiative is available at www.strobe-statement.org.

For peer review only

Spatial variation of Heart Failure and Air Pollution in Warwickshire, UK: An investigation of small scale variation at the ward-level

Journal:	*BMJ Open*
Manuscript ID:	bmjopen-2014-006028.R2
Article Type:	Research
Date Submitted by the Author:	03-Oct-2014
Complete List of Authors:	Bennett, Oscar; Warwickshire County Council, Public Health Warwickshire,Communities Group<br>Kandala, Ngianga-Bakwin; University of Warwick, Warwick Medical School<br>Ji, Chen; University of Warwick, Warwick Medical School<br>Linnane, John; Warwickshire County Council, Public Health Warwickshire,Communities Group<br>Clarke, Aileen; University of Warwick, Division of Health Sciences
**Primary Subject Heading**:	Epidemiology
Secondary Subject Heading:	Cardiovascular medicine, Epidemiology, Public health
Keywords:	Heart failure < CARDIOLOGY, EPIDEMIOLOGY, SOCIAL MEDICINE

SCHOLARONE™ Manuscripts

Spatial variation of Heart Failure and Air Pollution in Warwickshire, UK: An investigation of small scale variation at the ward-level

Oscar Bennett MB BChir[1], *Ngianga-Bakwin Kandala PhD*[2, 3], *Chen Ji PhD*[2], *John Linnane MD* FFPHM[1], *Aileen Clarke MD PhD*[2]

[1] Public Health Warwickshire, Communities Group, Warwickshire County Council ,Shire Hall, Barrack Street, Warwick, CV34 4RL, UK.
[2] Division of Health Sciences, University of Warwick Medical School, Coventry, CV4 7AL, UK.
[3] Division of Epidemiology and Biostatistics, School of Public Health, University of Witwatersrand, Johannesburg , South Africa.

Running title: Heart Failure and Air Pollution in Warwickshire

Keywords: Warwickshire, Heart Failure, Air Pollution, epidemiology, spatial analyses

Correspondence to:

Ngianga-Bakwin Kandala, PhD
Division of Health Sciences
University of Warwick Medical School
Medical School Building
Gibbet Hill Campus
Coventry CV4 7AL
United Kingdom
Tel: + 44 (0) 2476150541
Fax:+ 44 (0) 2476528375
Email: N-B.Kandala@warwick.ac.uk

For peer review only

For peer review only - http://bmjopen.bmj.com/site/about/guidelines.xhtml

Abstract

Objectives: To map using geospatial modelling techniques the morbidity and mortality caused by heart failure within Warwickshire to characterise and quantify any influence of air pollution on these risks.

Design: Cross-sectional.

Setting: Warwickshire, United Kingdom

Participants: Data from all of the 105 current Warwickshire County wards were collected on hospital admissions and deaths due to heart failure.

Results

In multivariate analyses, the presence of a higher Mono-nitrogen Oxide (NOx) in a ward [3.35:1.89, 4.99], Benzene (Ben) [31.9:8.36, 55.85] and Index of Multiple Deprivation (IMD) [0.02: 0.01, 0.03], were consistently associated with a higher risk of heart failure morbidity. Particulate matter (Pm) [-12.93: -20.41, -6.54] was negatively associated with the risk of heart failure morbidity. No association was found between sulphur dioxide (So2) and heart failure morbidity.

The risk of heart failure mortality was higher in wards with a higher NOx [4.30: 1.68, 7.37] and wards with more inhabitants 50+ years old [1.60: 0.47, 2.92]. Pm was negatively associated [-14.69: -23.46, -6.50] with heart failure mortality. So2, Ben, and IMD score were not associated with heart failure mortality.

There was a prominent variation in heart failure morbidity and mortality risk across wards, the highest risk being in the regions around Nuneaton and Bedworth.

Conclusion

This study showed distinct spatial patterns in heart failure morbidity and mortality, suggesting the potential role of environmental factors beyond individual-level risk factors. Air pollution levels should therefore be taken into account when considering the wider determinants of public health and the impact that changes in air pollution might have on the health of a population.

For peer review only

ARTICLE FOCUS

- Air pollution has been linked to the development and exacerbation of a number of health problems including cardiovascular diseases such as heart failure.
- Heart failure is a serious condition that unfortunately affects many people in the UK. This link seems especially clear for cardiovascular and respiratory diseases such as ischaemic heart disease, heart failure, asthma, influenza, and lung cancer
- It is well established that the ambient levels of air pollution in a region can have an impact on the health status of the population which inhabits it

KEY MESSAGES

1. Using a novel approach, this study has characterised and quantified the potential role of environmental factors (air pollutants) at the county-level.
2. The presence of a higher Mono-nitrogen Oxide (NOx) in a ward, benzene and poor Index of Multiple Deprivation (IMD) score were consistently associated with a higher risk of heart failure morbidity. Particulate matter (Pm) was negatively associated with the risk of heart failure morbidity.
3. The risk of heart failure mortality was higher in wards with a higher NOx and wards with more inhabitants 50+ years old. Pm was negatively associated with heart failure mortality.
4. Air pollution overall, when all pollution components were brought together into a combined index, was positively associated with both heart failure morbidity and mortality across Warwickshire.
5. There was a striking variation in heart failure morbidity and mortality risk across wards, the highest risk being in the regions around Nuneaton and Bedworth.

STRENGTHS AND LIMITATIONS

- The model employed a fully Bayesian approach using Markov Chain Monte Carlo (MCMC) techniques for inference and model checking [11, 12]

- A single air pollution measurement in 2010 was used and it was assumed that there was no significant change in this value over the 2005-2013 periods that mortality and hospital admission data was gathered from.
- The cross-sectional nature of the study unfortunately does not allow us to establish temporality and thus clearly demonstrate causality of the observed associations.
- This was an ecological study, analysing characteristics and risk at the ward rather than individual level. Consequently associations and conclusions found from the aggregate data may not be directly applicable to individuals.

For peer review only - http://bmjopen.bmj.com/site/about/guidelines.xhtml

Introduction

Air pollution has been linked to the development and exacerbation of a number of health problems. This link seems especially clear for cardiovascular and respiratory diseases such as ischaemic heart disease, heart failure, asthma, influenza, and lung cancer [1, 2, 3, 4]. It is well established that the ambient levels of air pollution in a region can have an impact on the health status of the population which inhabits it [5]. Air pollution levels should therefore be taken into account when considering the wider determinants of public health and the impact that changes in air pollution might have on the health of a population.

Warwickshire is an English county within the United Kingdom approximately 112 Kilometres North-West of London. The issue of air pollution has been highlighted in Warwickshire recently by the setting up of Air Quality Management Areas (AQMAs) in a number of different parts of the county. These are specific areas in the county that have been identified as places where, without a focused local council strategy to reduce air pollution, future government targets for air pollutant concentrations may not be met. Within Warwickshire the specific air pollutant that has been identified as a problem and led to these special areas being setup is mono-nitrogen oxides (NOx)[6].

The objective of the present study was therefore to look for any relationship between levels of air pollution and the morbidity and mortality associated with heart failure within Warwickshire in the last few years (2005-2013 for morbidity, 2007-2012 for mortality). We examined a range of traditional air pollutants for an ecological association with heart failure morbidity and mortality at the county level. Furthermore, the present analysis attempted to highlight spatial patterns in heart failure morbidity and mortality risk within the county, after multiple adjustments for proximate, county-level factors. The geographic locations of wards within the county can be considered as proxy measures of many other unmeasured factors such as availability and access to health services, individual health seeking behaviour,

For peer review only - http://bmjopen.bmj.com/site/about/guidelines.xhtml

preventive ward policy and general ward factors. Such estimates might illustrate how much can be learned by detailed exploratory analyses as well as how these data can be used to strategically inform policy aiming at the prevention and management of serious conditions such as heart failure in these settings.

The evidence from published literature for the link between air pollution and health problems

Studies have shown an effect on the health of populations caused by both long term exposure as well as short term "spikes" in local air pollution levels[5].

A relevant systematic review and meta-analysis has been published recently in the Lancet [2]. It pooled the results of studies that have been conducted worldwide looking at the temporal relationship between the levels of a number of different air pollutants with local heart failure hospital admission rates and mortality rates. This showed a clear link between short term rises in all types of air pollution (except ozone) and rises in hospital admissions and mortality due to heart failure. This study did not look at any potential effects from long term exposure to air pollution. However it did provide strong evidence that air pollution can 'exacerbate' heart failure, increasing the likelihood that a patient with existing heart failure will become sufficiently unwell to require hospital admission, or even die.

The ESCAPE study[3], published recently in the Lancet Oncology, pooling the results of 17 cohort studies from around Europe looked at the ambient levels of air pollution in an area (particulate matter and NOx) and the incidence of lung cancers in the inhabitants of this area during many years of follow up (mean 12.8 years). This was intended to look at the risk associated with long term exposure to air pollution. A statistically significant correlation was found between the levels of particulate matter air pollution and the local incidence of lung cancer diagnoses. Particulate matter with a diameter of less than 10 μm had a hazard ratio of 1.22 [95% CI 1.03-1.45] per (10 μg/cubic meter). In other words, for every increase in

For peer review only - http://bmjopen.bmj.com/site/about/guidelines.xhtml

particulate matter pollution of 10 μg/cubic meter there was a corresponding immediate increase in the chance of being diagnosed with lung cancer of 22% (95% CI 3% - 45%). There was also a correlation between traffic volume within 100m of a residence and a modest increase in the rate of lung cancer (Hazard ratio 1.09 (CI 0.99-1.21)). No similar correlation was found with NOx air pollution. This suggests that long term exposure to higher levels of particulate matter air pollution may increase the incidence of lung cancer in a population. Another study published in the United States in 2004 looked at the long term effect of exposure to particulate matter air pollution and the mortality attributed to different cardiovascular and respiratory diseases in different areas of the United States[(4)]. A good correlation was found between the degree of long term exposure to particulate matter air pollution and increases in mortality from cardiovascular diseases, including heart failure. Interestingly this was not found to be the case for most respiratory diseases. There was also an element of the study that looked at the effect of a person's smoking status on the mortality statistics. This found, as expected, a strong link between mortality and smoking. However it also found that air pollution contributed additional cardiovascular mortality risk on top of that attributed to smoking. This was at least an additive, if not a synergistic effect.

Methods

Study data

In order to carry out this project, data was collected about the geographical distribution of air pollution within Warwickshire. This data included information about each of four individual components of air pollution (NOx, sulphur dioxide, particulate matter, and benzene) which could then be united into a combined index (all of the contributions added together). A single recorded level from 2010 of each air pollutant for each ward was used in the study. This was then compared to collected data about:

For peer review only

(i) The geographical distribution of home addresses of patients who were admitted to hospital because of heart failure or a complication of heart failure. Hospital admission rate in an area due to heart failure was used as a proxy indicator for the level of heart failure morbidity within that area.

(ii) The geographical distribution of home addresses of patients who died from heart failure, or whose death was contributed to by heart failure.

These data were collected by the Warwickshire Observatory, which is part of the Warwickshire County Council in charge of collecting and handling statistics relating to the county. Mortality data for the analysis was supplied via the Warwickshire Public Health Intelligence Team and was sourced from the Public Health Mortality Files, Office for National Statistics. Hospital admissions data was accessed via the Ventris Business Intelligence System, Arden Commissioning Support Unit."

Ward level population data were obtained from the 2011 census. Warwickshire is divided into 105 wards. Data from all of the 105 current Warwickshire County wards were collected. The potential confounding variables of firstly age structure of a population within a ward and secondly levels of social deprivation within a ward were identified [7,8]. These were then controlled for in the second stage of the statistical analysis (see below).

Information about the age structure of wards was obtained from the Office of National Statistics mid 2010 estimates (obtained from the Warwickshire Observatory website)[9]. The representative statistical value of "percentage (%) of population above age 50" was used as an indicator of wards with a higher proportion of older people.

Information about social deprivation was obtained from the English Indices of Deprivation published by the Department for Communities and Local Government [10]. The Index of Multiple Deprivation (IMD) averaged across each ward was used as an indicator of the level of deprivation within the wards of Warwickshire.

For peer review only - http://bmjopen.bmj.com/site/about/guidelines.xhtml

The information on crude observed air pollution level distribution, heart failure hospital admission rates and mortality rates were then represented on maps of Warwickshire (**Figure 1**).

Statistical Analysis

To account for spatial autocorrelation in observed heart failure hospital admission and mortality rates at the ward level, in Warwickshire, we applied a unified approach to account for possible air pollution effects of environmental risk factors. This was achieved using a geo-additive semi-parametric mixed model. The model employed a fully Bayesian approach using Markov Chain Monte Carlo (MCMC) techniques for inference and model checking [11, 12]. Response variables were defined as the count (per 1000 population) of heart failure morbidity or mortality in a ward (Poisson model): $y_i /\eta_i,\delta \sim \mathrm{B}(\eta_i,\delta)$ for a binomial formulation.

(Poisson model): $y_i /\eta_i,\delta \sim \mathrm{B}(\eta_i,\delta)$ (1) for a binomial formulation and a geo-additive semi-parametric predictor $\mu_i = h(\eta_i)$:

$$\eta_i = f1(xi1) + ... + f_{p(xip)} + f_{spat}(s_i) + \varepsilon_i \qquad (2)$$

where h is a known response function with a poison link function, $f_1, ..., f_p$ are non-linear smoothed effects of the metrical covariates (time in years), and $f_{spat}(s_i)$ is the effect of the spatial covariate $s_i \in \{1, ..., S\}$ labelling the ward in Warwickshire. Regression models with predictors such as those in equation 2 are sometimes referred to as geo-additive models. P-spline priors were assigned to the functions $f1,...,f_p$, non-informative priors were used for fixed effects parameters and a Markov random field prior was used for $f_{spat}(s_i)$. More detailed information about the modelling approach can be found elsewhere [11, 13]. The standard measure of effect was the posterior mean (PM) and 95% credible region (CR).

The analysis was carried out using version 2.0.1 of the BayesX software package, which permits Bayesian inference based on MCMC simulation techniques[13]. Multivariate Bayesian geo-additive regression models were used to evaluate the significance of the posterior mean

For peer review only - http://bmjopen.bmj.com/site/about/guidelines.xhtml

(PM) determined for the fixed effects and spatial effects between air pollution and the morbidity and mortality from heart failure within Warwickshire. Each component of air pollution was looked at separately and then also the combined index in unadjusted models. Next, fully adjusted multivariate Bayesian geo-additive regressions analysis were performed to look again for statistically significant correlation between these variables, but this time further controlling for any influence from age structure or social deprivation.

Results

Figure 1 and **Figure 6** display the observed data collected for this study on maps using graduated colouring to represent data value categories within each ward.

Observed Air Quality Map

Figure 1left was produced using 2010 data from the Warwickshire Observatory. It is based on a combined air quality indicator which is a combination of information about the contribution to air pollution from NOx, sulphur dioxide, particulate matter, and benzene. The Warwickshire Observatory description of this index reads as follows:

"Combined Air Quality Indicator (estimates of emissions for four pollutants: benzene, nitrogen dioxide, sulphur dioxide and particulates) for small areas (modelled to 1 km grid squares) where an index value of 1 is equivalent to the national standard for each pollutant. The values are then summed so an overall score of 4 would represent all four pollutants being present at the national standard level."[(14)] These specific standards are described in the Air Quality Standards Regulations 2010.

The geographical pattern of the observed air pollution across the county shows a higher level of air pollution near the more urban centres of Birmingham and Coventry. The proximity of parts of the county to motorways such as the M6 and M42 could also be a contributing factor

For peer review only - http://bmjopen.bmj.com/site/about/guidelines.xhtml

For peer review only

to the observed pattern. There was no place in the county where the level of any pollutant exceeded the national standard in 2010.

Heart Failure Hospital Admission Map

Figure 1centre shows the geographical distribution of the density of home addresses of patients admitted to hospital with a diagnosis of heart failure (or exacerbation of heart failure) within the April 2005- April 2013 period.

Heart Failure Mortality Map

Figure 1right shows the geographical distribution of the density of home addresses of patients who died either directly or in part from heart failure in the 2007-2012 (inclusive) periods.

Table 1 (left panel) displays posterior means of heart failure admission across the selected covariates following multivariate Bayesian geo-additive regression analyses.

The Posterior mean (PM) & 95% Credible Region (CR) of overall hospital admission rates due to heart failure and mortality rates from heart failure were [6.19 (3.84, 8.97)] and [4.13 (1.18, 7.43)] (Table 1) respectively. On average, the presence of a higher Mono-nitrogen oxide indicator (NOx) in a ward [Posterior mean (PM) & 95% Credible Region (CR): 3.35 (1.89, 4.99)], Benzene indicator (Ben) [31.9 (8.36, 55.85)] and IMD 2010 score [0.02 (0.01, 0.03)], were consistently associated with higher risk of heart failure morbidity. The particulates indicator (Pm) [-12.93 (-20.41, -6.54)] was negatively associated with the risk of heart failure morbidity. Sulphur dioxide indicator (So2) was not associated with heart failure morbidity.

For peer review only - http://bmjopen.bmj.com/site/about/guidelines.xhtml

For peer review only

Table 1 (right panel) shows the same corresponding results for heart failure mortality. This table shows that the risk of heart failure mortality was higher in wards with a higher Mono-nitrogen oxide indicator (NOx) [4.30 (1.68, 7.37)] and wards with more inhabitants over 50 years old [1.60 (0.47, 2.92)]. The particulates indicator (Pm) was negatively associated with heart failure mortality. The sulphur dioxide indicator (So2), Benzene indicator (Ben), and IMD score were not associated with heart failure mortality.

For peer review only

The combined air pollution index (all indicators averaged together) when incorporated into a separate model combining age and social deprivation was significantly positively associated with both heart failure morbidity [1.39 (0.87, 1.81)] and mortality [1.79 (0.85, 2.55)] across the county.

In **Figure 2** and **Figure 4**, the left-hand maps show the unadjusted estimates of posterior total residual ward means of heart failure morbidity and mortality respectively. In **Figure 3** and **Figure 5**, the left-hand maps show the adjusted PM after multiple adjustment for the geographical location, taking into account the auto-correlation structure in the data, the uncertainty in the ward level and all ward-level risk factors (air pollution components, age, social deprivation) for heart failure morbidity and mortality respectively. The red colour indicates the maximum posterior mean recorded while green denotes the lowest mean. The right-hand maps in **Figures 2**, **3, 4** and **5** show the 95% posterior probability of heart failure and mortality, which indicate the statistical significance associated with the total excess risk. Black colour indicates a negative spatial effect (associated with increased risk of heart failure and mortality), white colour a positive effect (a decreased risk) and grey colour a non-significant effect.

In general, there was consistently higher heart failure morbidity risk in northern wards particularly around Nuneaton and Bedworth and lower heart failure morbidity risks in the

For peer review only - http://bmjopen.bmj.com/site/about/guidelines.xhtml

southern wards particularly within the district of Stratford-on-Avon. However, all this variation could partially be accounted for within the model generated within this study (taking into account air pollution, age, and social deprivation levels).

Heart failure mortality risk was, in general, higher in northern wards around Nuneaton and Bedworth as well as in some central areas around Warwick, Royal Leamington Spa, and Kenilworth. Heart failure mortality risk was again lower in more southern wards particularly within Stratford-on-Avon. Unlike with morbidity however, our model could not explain all this variation in heart failure mortality. Even after taking into account air pollution, age, and social deprivation the rate of death from heart failure remained significantly higher than would be expected in areas within and around Nuneaton, Bedworth, Warwick, Royal Leamington Spa, and Kenilworth.

In sensitivity analyses, we tested several models and our results were not substantially altered after removing one or two pollutants (data not shown).

Discussion

Before even considering air pollution, this study helps to demonstrate the inequality of risk from heart failure disease and death that exists for individuals living in different parts of the county of Warwickshire. There is a significant excess risk of both disease and death in more northern wards within and surrounding Nuneaton and Bedworth. Much (but not all) of this variation could be attributed to the air pollution, age structure and social deprivation that exists in these areas according to our model. Measures that seek to address air pollution and social deprivation could be expected therefore to help mitigate against cardiovascular risks within these local populations.

For peer review only - http://bmjopen.bmj.com/site/about/guidelines.xhtml

The present study corroborates the notion that air pollution is an increasingly important public health issue in Warwickshire. Higher levels of the average air pollution index looked at in this study correlated significantly with increased levels of both heart failure morbidity and mortality across the county, even after removing the effects of age structure and social deprivation. The individual component of NOx air pollution seems particularly to contribute risk to both the morbidity and mortality of heart failure within the county, suggesting that it may have a particularly detrimental effect on heart failure patients. This reinforces the importance of the AQMAs set up within the county in response to high NOx levels. Road traffic is a large contributor to air pollution within Warwickshire. Diesel engines are responsible for a large part of the NOx component of these emissions.

An unexpected result also appeared within our analysis. Particulate matter air pollution became significantly, negatively correlated with both heart failure morbidity and mortality when incorporated into our model with all risk factors taken into account and controlled for in this study. This would imply some sort of unexpected "protective influence" from particulate matter air pollution on heart failure patients. This clearly contradicts our expectations and is at odds with a wealth of existing evidence that indicates that particulate matter air pollution contributes risk to and exacerbates cardiovascular disease[(4)].

We offer a possible explanation for this based on the following four observations:

1. The above-mentioned negative correlation of particulate matter air pollution with heart failure morbidity and mortality in our model.
2. Particulate matter air pollution actually varied very little across the county compared to the other types of air pollution. All types of air pollution tended to decrease in rural areas, but particulate matter tended to decrease much *less* compared to the other components of air pollution. Consequently in rural areas of the county where most

For peer review only - http://bmjopen.bmj.com/site/about/guidelines.xhtml

types of air pollution are significantly lower, particulate matter pollution was relatively higher compared to NOx, Benzene, and SO2.

3. There seems to be a high risk of heart failure deaths in urban centres (particularly Nuneaton, Bedworth, Warwick, Royal Leamington Spa, and Kenilworth), higher than can be explained by our model.
4. Conversely, there seems to be a particularly low risk of heart failure deaths in some rural areas within the western part of Stratford-on-Avon, lower than can be explained by our model.

A possible hypothesis based on these observations is that there is an additional factor influencing the morbidity and mortality of heart failure not looked at in this study, namely the urban/rural nature of a patient's living environment. It could be the case that living in an urban environment contributed risk and living in a rural area provided protection against heart failure morbidity and mortality. This would be an effect in *addition* to any increase in air pollution or social deprivation within urban settings compared to rural settings. This could certainly in principle be plausible, with people in rural areas perhaps doing more physical activity, eating more healthily etc. If this were the case it would explain the excess deaths in urban centres found in this study. It could also be responsible for the unexpected protective factor attributed to particulate matter air pollution in our analysis. Given that the particulate matter component of air pollution is *relatively* higher than the other components in rural areas, the protection that living in rural areas affords individuals could be misleadingly attributed (in our statistical analysis) to the particulate matter component of air pollution present in these areas. Further work will need to be done looking into this possible link between urban/rural living environments and heart failure morbidity and mortality. It could be very revealing to carefully characterise this effect if it indeed exists, as it may be an indication of unrecognised cardiovascular risk/protective factors associated with urban/rural living that exists within Warwickshire.

For peer review only - http://bmjopen.bmj.com/site/about/guidelines.xhtml

For peer review only

However, it is also important to bear in mind that this is an ecological study and all the relationships picked up between variables in this study have been found using aggregate data at the ward level (number over 50 years of age, average IMD score, average air pollution across ward, overall numbers of deaths and hospital admissions due to heart failure). It is not always a trivial task to extrapolate the conclusions drawn from such a study down to the level of individuals. Such a task would involve drilling down to individual level data and repeating the analysis, a task that was beyond the scope of this particular study. It is possible that the unexpected negative correlation between particulate matter air pollution and heart failure could disappear when data is analysed at the individual level – an example of an ecological fallacy. Consequently it would be prudent to regard the results from the individual components of air pollution with cautious interest, rather than viewing them as proof of any real effects.

However, despite these caveats, this study has been able to provide some helpful information at the population level worthy of consideration. A health inequality has been revealed, and the manner with which this inequality is influenced by age, social deprivation, and the combined index of air pollution has been demonstrated. Such information should help inform policy decisions that would influence society at a population level and hopefully improve public health in the long run.

There are some limitations in this study worth considering that result from assumptions made along the way. A single air pollution measurement in 2010 was used and it was assumed that there was no significant change in this value over the 2005-2013 periods that mortality and hospital admission data was gathered from. The resulting cross-sectional nature of the study does not allow establishing temporality and thus causality of the observed associations. There was also no way to determine the length of time that individual members of the population within a ward had lived in that area, and thus how long they had been exposed to the

For peer review only - http://bmjopen.bmj.com/site/about/guidelines.xhtml

measured ambient air pollution level. It was assumed that people with home addresses in a ward were exposed significantly to the levels of air pollution in that ward. Finally, as already mentioned, this was an ecological study, using aggregate data of risk factors to look for associations with aggregate data of morbidity and mortality. It is not always possible to apply such associations from a population level down to the individuals within that population.

In summary, this study has provided a number of interesting results. Firstly it has helped to quantify and map the inequality that exists across different parts of Warwickshire with regards to heart failure risk. It has also provided some interesting circumstantial evidence of a link between heart failure morbidity and air pollution. Finally it has also given a suggestion of a possible link between living in urban environments and a higher risk of cardiovascular disease, and a corresponding lower risk from living in rural environments. More work will need to be done to look into this particular possibility. It would be informative to run this type of analysis whilst factoring in the influence of a person's distance from their nearest urban centre. This urban/rural factor should be further explored and mined for additional information as it could be an indication of hitherto unconsidered factors influencing the health status of the population of Warwickshire and possibly further afield.

In order to determine the validity of our conclusions at the individual level further work would need to be done analysing the available data from individual patients (risks and outcomes). Such work could help to characterise the true effect of different components of air pollution at the individual level. It would also be interesting to determine if the different components of air pollution act as effect modifiers on each other.

It would be possible to look at the effects of air pollution variation in the shorter term as well. For example, looking at how local "spikes" in air pollution affect the rates of hospital admissions locally immediately following it. This could be done in Leamington Spa where there is an air quality monitoring station constantly measuring the levels of air pollutants.

For peer review only - http://bmjopen.bmj.com/site/about/guidelines.xhtml

Other health problems, such as ischaemic heart disease and respiratory diseases, have been linked with air pollution as well and it could be informative to also look into these links locally.

Contributorship statement:

OB conceived the idea, analyzed the data, contributed to formulating the results and wrote the first draft. N-B K analyzed the data, advised on statistical aspects, contributed to formulating the results, wrote the second draft. CJ analyzed the data. JL helped coordinate the project and co-wrote the final draft; AC coordinated the project, advised on all aspects and co-wrote the final draft.

Funding:

This paper presents independent research supported by the National Institute for Health Research (NIHR) Collaborations for Leadership in Applied Health Research and Care West Midlands. The views expressed are those of the author(s) and not necessarily those of the NHS, the NIHR or the Department of Health.

Competing interests: The authors declared no conflict of interest.

Data sharing: No additional data available.

References

1. Particulate Air Pollution and Hospital Emergency Room Visits for Asthma in Seattle, Joel Schwartz, Daniel Slater, Timothy V. Larson, William E. Pierson, and Jane Q. Koenig. American Review of Respiratory Disease, Vol. 147, No. 4 (1993), pp. 826-831.
2. Global association of air pollution and heart failure: a systematic review and meta-analysis, Anoop SV Shah, Jeremy P Langrish,Harish Nair,David A McAllister, Amanda L Hunter, Prof Ken Donaldson, Prof David E Newby, Dr Nicholas L Mills. The Lancet, Early Online Publication, 10 July 2013 DOI: 10.1016/S0140-6736(13)60898-3
3. Air pollution and lung cancer incidence in 17 European cohorts: prospective analyses from the European Study of Cohorts for Air Pollution Effects (ESCAPE), Raaschou-Nielsen O, Andersen ZJ, et al. Lancet Oncol. 2013 Aug;14(9):813-22
4. Cardiovascular Mortality and Long-Term Exposure to Particulate Air Pollution, Epidemiological Evidence of General Pathophysiological Pathways of Disease C. Arden Pope III, Richard T. Burnett, et al. Circulation. 2004; 109: 71-77

For peer review only - http://bmjopen.bmj.com/site/about/guidelines.xhtml

5. Air pollution and health, Bert Brunekreef, Stephen T Holgate, The Lancet, Volume 360, Issue 9341, 19 October 2002, Pages 1233–1242
6. Department of Environment, Food and Rural Affairs (DEFRA), department website, weblink (accessed 2/12/13): http://aqma.defra.gov.uk/aqma/local_authorities.php?la_id=296
7. Epidemiology, pathophysiology, and etiology of congestive heart failure in older adults. Rich MW. J Am Geriatr Soc. 1997 Aug;45(8):968-74.
8. Influence of socioeconomic deprivation on the primary care burden and treatment of patients with a diagnosis of heart failure in general practice in Scotland: population based study. F A McAlister, N F Murphy, et al. BMJ. 2004 May 8; 328(7448): 1110.
9. Warwickshire Observatory website – ONS population estimates. Web link (accessed on 3/10/13): http://www.warwickshireobservatory.org/observatory/observatorywcc.nsf/05a17cfbd3bcf85d802572920033cf0f/bfb8b0a1d34f274c802572c00048108f?OpenDocument
10. Deprivation data produced by the Department for Communities and Local Government. File located at web link (accessed on 3/10/13): https://www.gov.uk/government/publications/english-indices-of-deprivation-2010 Ward based population weighted averages calculated from LSOA specific data by the London Health Observatory and North East Public Health Observatory.
11. Fahrmeir L, Lang S. Bayesian Inference for Generalized Additive Mixed Models Based on Markov Random Field Priors. *Applied Statistics (JRSS C)*. 2001;50:201-20.
12. Kandala N-B, Tigbe W, Manda S, Stranges S. Spatial Variation of Predictors of Hypertension in Sub-Saharan Africa: A Case Study of South-Africa. American Journal of Hypertension 2013. 10.1093/ajh/hps063.
13. Belitz C, Brezger A, Kneib T, Lang S. BayesX Software for Bayesian Inference in Structured Additive Regression Models Version 2.0.1, 2012. Accessed at: http://www.stat.uni-muenchen.de/~bayesx/manual/methodology_manual.pdf
14. Taken from the Warwickshire Observatory Quality of Life in Warwickshire 2012/13 report. Weblink (accessed on 3/10/13): http://maps.warwickshire.gov.uk/iareports/environmental_issues/

Author's contributions

OB conceived the idea, analyzed the data, contributed to formulating the results and wrote the first draft.

N-B K analyzed the data, advised on statistical aspects, contributed to formulating the results, wrote the second draft. CJ analyzed the data. JL helped coordinate the project and co-wrote the final draft; AC coordinated the project, advised on all aspects and co-wrote the final draft.

For peer review only - http://bmjopen.bmj.com/site/about/guidelines.xhtml

Figure legends

Figure 1 : Warwickshire map with 2010 air quality index (all components of air pollution combined) displayed by ward (left; Warwickshire map with number of heart failure hospital admissions per 1000 population between 2005 – 2013 displayed by ward(centre) and Warwickshire map with total heart failure deaths per 1000 population between 2007 – 2012 displayed by ward(right).

[FIGURE 1 ABOUT HERE]

Figure 2: Left: Unadjusted total residual spatial effects of morbidity risk associated with heart failure, at ward level in Warwickshire. Shown are the posterior means. Right: Corresponding posterior probabilities at 80% nominal level.

[FIGURE 2 ABOUT HERE]

Red coloured – high risk
Green coloured – low risk

Black coloured – significant positive spatial effect
White coloured- significant negative spatial effect
Grey coloured – no significant effect

Figure 3: Left: Total residual spatial effects of morbidity risk associated with heart failure, at ward level in Warwickshire. Shown are the posterior means of the full model (IMD 2010, Over 50, and the 4 indicators of air pollution). Right: Corresponding posterior probabilities at 80% nominal level.

[FIGURE 3 ABOUT HERE]

Red coloured – high risk
Green coloured – low risk

Black coloured – significant positive spatial effect
White coloured- significant negative spatial effect
Grey coloured – no significant effect

Figure 4: Left: Unadjusted total residual spatial effects of mortality risk associated with heart failure, at ward level in Warwickshire. Shown are the posterior means. Right: Corresponding posterior probabilities at 80% nominal level.

[FIGURE 4 ABOUT HERE]

Red coloured – high risk
Green coloured – low risk

Black coloured – significant positive spatial effect
White coloured- significant negative spatial effect
Grey coloured – no significant effect

Figure 5: Left: Total residual spatial effects of mortality risk associated with heart failure, at ward level in Warwickshire. Shown are the posterior means of the full model (IMD 2010, Over 50, and the 4 indicators of air pollution). Right: Corresponding posterior probabilities at 80% nominal level.

[FIGURE 5 ABOUT HERE]

Red coloured – high risk
Green coloured – low risk

Black coloured – significant positive spatial effect
White coloured- significant negative spatial effect
Grey coloured – no significant effect

Figure 6 – Warwickshire map displaying the 2010 levels of deprivation (expressed as Multiple Deprivation Scores) by LSOAs. Produced by the Warwickshire Observatory (left) and Warwickshire map displaying the 2008 midyear estimates of % of people over the age of 50 by SOA. Produced by the Warwickshire Observatory (right).

For peer review only - http://bmjopen.bmj.com/site/about/guidelines.xhtml

Table 1: Posterior mean (PM) of fixed effects estimates of heart failure admission and mortality across air pollutions indicators (Warwickshire 2005-2013)

**Variable**	Heart failure admission PM & 95%CI‡	Heart failure mortality PM & 95%CI †
Constant	6.19 (3.84, 8.97)	4.13 (1.18, 7.43)
Nitrogen dioxide indicator (No2)	3.35 (1.89, 4.99)	4.30 (1.68, 7.37)
Sulphur dioxide indicator (So2))	7.75 (-3.84, 17.99)	11.02 (-1.21, 22.41)
Particulates indicator (Pm)	-12.93 (-20.41, -6.54)	-14.69 (-23.46, -6.50)
Benzene indicator (Ben)	[31.9:8.36, 55.85]	25.98 (-6.62, 54.22)
Over 50 years %	0.70(-0.63, 1.98)	1.60, (0.47, 2.92)
IMD 2010 score	0.02 (0.01, 0.03)	0.00 (-0.01, 0.01)

‡ Spatially adjusted posterior mean (PM) from Bayesian geo-additive regression models after controlling for fixed effect of all air pollutions indicators: Mono-nitrogen Oxide (NOx), sulphur dioxide (So2), particulate matter (Pm), benzene (Ben) and combined index and the county of residence (spatial effects). †Spatially adjusted posterior mean (PM) from Bayesian geo-additive regression models after controlling for fixed effect of all air pollutions indicators: Mono-nitrogen Oxide (NOx), sulphur dioxide (So2), particulate matter (Pm), benzene (Ben) and combined index and the county of residence (spatial effects).

For peer review only

For peer review only - http://bmjopen.bmj.com/site/about/guidelines.xhtml

Spatial variation of Heart Failure and Air Pollution in Warwickshire, UK: An investigation of small scale variation at the ward-level

Oscar Bennett MB BChir[1], *Ngianga-Bakwin Kandala PhD*[2, 3], *Chen Ji PhD*[2], *John Linnane MD* FFPHM[1], *Aileen Clarke MD PhD*[2]

[1] Public Health Warwickshire, Communities Group, Warwickshire County Council ,Shire Hall, Barrack Street, Warwick, CV34 4RL, UK.
[2] Division of Health Sciences, University of Warwick Medical School, Coventry, CV4 7AL, UK.
[3] Division of Epidemiology and Biostatistics, School of Public Health, University of Witwatersrand, Johannesburg , South Africa.

Running title: Heart Failure and Air Pollution in Warwickshire

Keywords: Warwickshire, Heart Failure, Air Pollution, epidemiology, spatial analyses

Correspondence to:

Ngianga-Bakwin Kandala, PhD
Division of Health Sciences
University of Warwick Medical School
Medical School Building
Gibbet Hill Campus
Coventry CV4 7AL
United Kingdom
Tel: + 44 (0) 2476150541
Fax:+ 44 (0) 2476528375
Email: N-B.Kandala@warwick.ac.uk

For peer review only - http://bmjopen.bmj.com/site/about/guidelines.xhtml

Abstract

Objectives: To map using geospatial modelling techniques the morbidity and mortality caused by heart failure within Warwickshire to characterise and quantify any influence of air pollution on these risks.

Design: Cross-sectional.

Setting: Warwickshire, United Kingdom

Participants: Data from all of the 105 current Warwickshire County wards were collected on hospital admissions and deaths due to heart failure.

Results

In multivariate analyses, the presence of a higher Mono-nitrogen Oxide (NOx) in a ward [3.35:1.89, 4.99], Benzene (Ben) [31.9:8.36, 55.85] and Index of Multiple Deprivation (IMD) [0.02: 0.01, 0.03], were consistently associated with a higher risk of heart failure morbidity. Particulate matter (Pm) [-12.93: -20.41, -6.54] was negatively associated with the risk of heart failure morbidity. No association was found between sulphur dioxide (So2) and heart failure morbidity.

The risk of heart failure mortality was higher in wards with a higher NOx [4.30: 1.68, 7.37] and wards with more inhabitants 50+ years old [1.60: 0.47, 2.92]. Pm was negatively associated [-14.69: -23.46, -6.50] with heart failure mortality. So2, Ben, and IMD score were not associated with heart failure mortality.

There was a prominent variation in heart failure morbidity and mortality risk across wards, the highest risk being in the regions around Nuneaton and Bedworth.

Conclusion

This study showed distinct spatial patterns in heart failure morbidity and mortality, suggesting the potential role of environmental factors beyond individual-level risk factors. Air pollution levels should therefore be taken into account when considering the wider determinants of public health and the impact that changes in air pollution might have on the health of a population.

For peer review only - http://bmjopen.bmj.com/site/about/guidelines.xhtml

ARTICLE FOCUS

- Air pollution has been linked to the development and exacerbation of a number of health problems including cardiovascular diseases such as heart failure.
- Heart failure is a serious condition that unfortunately affects many people in the UK. This link seems especially clear for cardiovascular and respiratory diseases such as ischaemic heart disease, heart failure, asthma, influenza, and lung cancer.
- It is well established that the ambient levels of air pollution in a region can have an impact on the health status of the population which inhabits it

KEY MESSAGES

1. Using a novel approach, this study has characterised and quantified the potential role of environmental factors (air pollutants) at the county-level.
2. The presence of a higher Mono-nitrogen Oxide (NOx) in a ward, benzene and poor Index of Multiple Deprivation (IMD) score were consistently associated with a higher risk of heart failure morbidity. Particulate matter (Pm) was negatively associated with the risk of heart failure morbidity.
3. The risk of heart failure mortality was higher in wards with a higher NOx and wards with more inhabitants 50+ years old. Pm was negatively associated with heart failure mortality.
4. Air pollution overall, when all pollution components were brought together into a combined index, was positively associated with both heart failure morbidity and mortality across Warwickshire.
5. There was a striking variation in heart failure morbidity and mortality risk across wards, the highest risk being in the regions around Nuneaton and Bedworth.

STRENGTHS AND LIMITATIONS

- The model employed a fully Bayesian approach using Markov Chain Monte Carlo (MCMC) techniques for inference and model checking [11, 12]

- A single air pollution measurement in 2010 was used and it was assumed that there was no significant change in this value over the 2005-2013 periods that mortality and hospital admission data was gathered from.
- The cross-sectional nature of the study unfortunately does not allow us to establish temporality and thus clearly demonstrate causality of the observed associations.
- This was an ecological study, analysing characteristics and risk at the ward rather than individual level. Consequently associations and conclusions found from the aggregate data may not be directly applicable to individuals.

For peer review only - http://bmjopen.bmj.com/site/about/guidelines.xhtml

Introduction

Air pollution has been linked to the development and exacerbation of a number of health problems. This link seems especially clear for cardiovascular and respiratory diseases such as ischaemic heart disease, heart failure, asthma, influenza, and lung cancer [1, 2, 3, 4]. It is well established that the ambient levels of air pollution in a region can have an impact on the health status of the population which inhabits it [5]. Air pollution levels should therefore be taken into account when considering the wider determinants of public health and the impact that changes in air pollution might have on the health of a population.

Warwickshire is an English county within the United Kingdom approximately 112 Kilometres North-West of London. The issue of air pollution has been highlighted in Warwickshire recently by the setting up of Air Quality Management Areas (AQMAs) in a number of different parts of the county. These are specific areas in the county that have been identified as places where, without a focused local council strategy to reduce air pollution, future government targets for air pollutant concentrations may not be met. Within Warwickshire the specific air pollutant that has been identified as a problem and led to these special areas being setup is mono-nitrogen oxides (NOx)[6].

The objective of the present study was therefore to look for any relationship between levels of air pollution and the morbidity and mortality associated with heart failure within Warwickshire in the last few years (2005-2013 for morbidity, 2007-2012 for mortality). We examined a range of traditional air pollutants for an ecological association with heart failure morbidity and mortality at the county level. Furthermore, the present analysis attempted to highlight spatial patterns in heart failure morbidity and mortality risk within the county, after multiple adjustments for proximate, county-level factors. The geographic locations of wards within the county can be considered as proxy measures of many other unmeasured factors such as availability and access to health services, individual health seeking behaviour,

For peer review only - http://bmjopen.bmj.com/site/about/guidelines.xhtml

preventive ward policy and general ward factors. Such estimates might illustrate how much can be learned by detailed exploratory analyses as well as how these data can be used to strategically inform policy aiming at the prevention and management of serious conditions such as heart failure in these settings.

The evidence from published literature for the link between air pollution and health problems

Studies have shown an effect on the health of populations caused by both long term exposure as well as short term "spikes" in local air pollution levels[5].

A relevant systematic review and meta-analysis has been published recently in the Lancet [2]. It pooled the results of studies that have been conducted worldwide looking at the temporal relationship between the levels of a number of different air pollutants with local heart failure hospital admission rates and mortality rates. This showed a clear link between short term rises in all types of air pollution (except ozone) and rises in hospital admissions and mortality due to heart failure. This study did not look at any potential effects from long term exposure to air pollution. However it did provide strong evidence that air pollution can 'exacerbate' heart failure, increasing the likelihood that a patient with existing heart failure will become sufficiently unwell to require hospital admission, or even die.

The ESCAPE study[3], published recently in the Lancet Oncology, pooling the results of 17 cohort studies from around Europe looked at the ambient levels of air pollution in an area (particulate matter and NOx) and the incidence of lung cancers in the inhabitants of this area during many years of follow up (mean 12.8 years). This was intended to look at the risk associated with long term exposure to air pollution. A statistically significant correlation was found between the levels of particulate matter air pollution and the local incidence of lung cancer diagnoses. Particulate matter with a diameter of less than 10 μm had a hazard ratio of 1.22 [95% CI 1.03-1.45] per (10 μg/cubic meter). In other words, for every increase in

For peer review only - http://bmjopen.bmj.com/site/about/guidelines.xhtml

particulate matter pollution of 10 μg/cubic meter there was a corresponding immediate increase in the chance of being diagnosed with lung cancer of 22% (95% CI 3% - 45%). There was also a correlation between traffic volume within 100m of a residence and a modest increase in the rate of lung cancer (Hazard ratio 1.09 (CI 0.99-1.21)). No similar correlation was found with NOx air pollution. This suggests that long term exposure to higher levels of particulate matter air pollution may increase the incidence of lung cancer in a population. Another study published in the United States in 2004 looked at the long term effect of exposure to particulate matter air pollution and the mortality attributed to different cardiovascular and respiratory diseases in different areas of the United States[(4)]. A good correlation was found between the degree of long term exposure to particulate matter air pollution and increases in mortality from cardiovascular diseases, including heart failure. Interestingly this was not found to be the case for most respiratory diseases. There was also an element of the study that looked at the effect of a person's smoking status on the mortality statistics. This found, as expected, a strong link between mortality and smoking. However it also found that air pollution contributed additional cardiovascular mortality risk on top of that attributed to smoking. This was at least an additive, if not a synergistic effect.

Methods

Study data

In order to carry out this project, data was collected about the geographical distribution of air pollution within Warwickshire. This data included information about each of four individual components of air pollution (NOx, sulphur dioxide, particulate matter, and benzene) which could then be united into a combined index (all of the contributions added together). A single recorded level from 2010 of each air pollutant for each ward was used in the study. This was then compared to collected data about:

For peer review only - http://bmjopen.bmj.com/site/about/guidelines.xhtml

(i) The geographical distribution of home addresses of patients who were admitted to hospital because of heart failure or a complication of heart failure. Hospital admission rate in an area due to heart failure was used as a proxy indicator for the level of heart failure morbidity within that area.

(ii) The geographical distribution of home addresses of patients who died from heart failure, or whose death was contributed to by heart failure.

These data were collected by the Warwickshire Observatory, which is part of the Warwickshire County Council in charge of collecting and handling statistics relating to the county. Mortality data for the analysis was supplied via the Warwickshire Public Health Intelligence Team and was sourced from the Public Health Mortality Files, Office for National Statistics. Hospital admissions data was accessed via the Ventris Business Intelligence System, Arden Commissioning Support Unit."

Ward level population data were obtained from the 2011 census. Warwickshire is divided into 105 wards. Data from all of the 105 current Warwickshire County wards were collected. The potential confounding variables of firstly age structure of a population within a ward and secondly levels of social deprivation within a ward were identified [7,8]. These were then controlled for in the second stage of the statistical analysis (see below).

Information about the age structure of wards was obtained from the Office of National Statistics mid 2010 estimates (obtained from the Warwickshire Observatory website)[9]. The representative statistical value of “percentage (%) of population above age 50” was used as an indicator of wards with a higher proportion of older people.

Information about social deprivation was obtained from the English Indices of Deprivation published by the Department for Communities and Local Government [10]. The Index of Multiple Deprivation (IMD) averaged across each ward was used as an indicator of the level of deprivation within the wards of Warwickshire.

For peer review only - http://bmjopen.bmj.com/site/about/guidelines.xhtml

The information on crude observed air pollution level distribution, heart failure hospital admission rates and mortality rates were then represented on maps of Warwickshire (**Figure 1**).

Statistical Analysis

To account for spatial autocorrelation in observed heart failure hospital admission and mortality rates at the ward level, in Warwickshire, we applied a unified approach to account for possible air pollution effects of environmental risk factors. This was achieved using a geo-additive semi-parametric mixed model. The model employed a fully Bayesian approach using Markov Chain Monte Carlo (MCMC) techniques for inference and model checking [11, 12]. Response variables were defined as the count (per 1000 population) of heart failure morbidity or mortality in a ward (Poisson model): $y_i / \eta_i, \delta \sim B(\eta_i, \delta)$ for a binomial formulation.

(Poisson model): $y_i / \eta_i, \delta \sim B(\eta_i, \delta)$ (1) for a binomial formulation and a geo-additive semi-parametric predictor $\mu_i = h(\eta_i)$:

$$\eta_i = f1(xi1) + ... + f_{p(xip)} + f_{spat}(s_i) + \varepsilon_i \qquad (2)$$

where h is a known response function with a poison link function, f_1, ..., f_p are non-linear smoothed effects of the metrical covariates (time in years), and $f_{spat}(s_i)$ is the effect of the spatial covariate $s_i \in \{1, ..., S\}$ labelling the ward in Warwickshire. Regression models with predictors such as those in equation 2 are sometimes referred to as geo-additive models. P-spline priors were assigned to the functions $f1, ..., f_p$, non-informative priors were used for fixed effects parameters and a Markov random field prior was used for $f_{spat}(s_i)$. More detailed information about the modelling approach can be found elsewhere [11, 13]. The standard measure of effect was the posterior mean (PM) and 95% credible region (CR).

The analysis was carried out using version 2.0.1 of the BayesX software package, which permits Bayesian inference based on MCMC simulation techniques[13]. Multivariate Bayesian geo-additive regression models were used to evaluate the significance of the posterior mean

For peer review only - http://bmjopen.bmj.com/site/about/guidelines.xhtml

(PM) determined for the fixed effects and spatial effects between air pollution and the morbidity and mortality from heart failure within Warwickshire. Each component of air pollution was looked at separately and then also the combined index in unadjusted models. Next, fully adjusted multivariate Bayesian geo-additive regressions analysis were performed to look again for statistically significant correlation between these variables, but this time further controlling for any influence from age structure or social deprivation.

Results

Figure 1 and **Figure 6** display the observed data collected for this study on maps using graduated colouring to represent data value categories within each ward.

Observed Air Quality Map

Figure 1left was produced using 2010 data from the Warwickshire Observatory. It is based on a combined air quality indicator which is a combination of information about the contribution to air pollution from NOx, sulphur dioxide, particulate matter, and benzene. The Warwickshire Observatory description of this index reads as follows:

"Combined Air Quality Indicator (estimates of emissions for four pollutants: benzene, nitrogen dioxide, sulphur dioxide and particulates) for small areas (modelled to 1 km grid squares) where an index value of 1 is equivalent to the national standard for each pollutant. The values are then summed so an overall score of 4 would represent all four pollutants being present at the national standard level."[(14)] These specific standards are described in the Air Quality Standards Regulations 2010.

The geographical pattern of the observed air pollution across the county shows a higher level of air pollution near the more urban centres of Birmingham and Coventry. The proximity of parts of the county to motorways such as the M6 and M42 could also be a contributing factor

For peer review only - http://bmjopen.bmj.com/site/about/guidelines.xhtml

to the observed pattern. There was no place in the county where the level of any pollutant exceeded the national standard in 2010.

Heart Failure Hospital Admission Map

Figure 1centre shows the geographical distribution of the density of home addresses of patients admitted to hospital with a diagnosis of heart failure (or exacerbation of heart failure) within the April 2005- April 2013 period.

Heart Failure Mortality Map

Figure 1right shows the geographical distribution of the density of home addresses of patients who died either directly or in part from heart failure in the 2007-2012 (inclusive) periods.

Table 1 (left panel) displays posterior means of heart failure admission across the selected covariates following multivariate Bayesian geo-additive regression analyses.

The Posterior mean (PM) & 95% Credible Region (CR) of overall hospital admission rates due to heart failure and mortality rates from heart failure were [6.19 (3.84, 8.97)] and [4.13 (1.18, 7.43)] (Table 1) respectively. On average, the presence of a higher Mono-nitrogen oxide indicator (NOx) in a ward [Posterior mean (PM) & 95% Credible Region (CR): 3.35 (1.89, 4.99)], Benzene indicator (Ben) [31.9 (8.36, 55.85)] and IMD 2010 score [0.02 (0.01, 0.03)], were consistently associated with higher risk of heart failure morbidity. The particulates indicator (Pm) [-12.93 (-20.41, -6.54)] was negatively associated with the risk of heart failure morbidity. Sulphur dioxide indicator (So2) was not associated with heart failure morbidity.

For peer review only - http://bmjopen.bmj.com/site/about/guidelines.xhtml

Table 1 (right panel) shows the same corresponding results for heart failure mortality. This table shows that the risk of heart failure mortality was higher in wards with a higher Mono-nitrogen oxide indicator (NOx) [4.30 (1.68, 7.37)] and wards with more inhabitants over 50 years old [1.60 (0.47, 2.92)]. The particulates indicator (Pm) was negatively associated with heart failure mortality. The sulphur dioxide indicator (So2), Benzene indicator (Ben), and IMD score were not associated with heart failure mortality.

For peer review only

The combined air pollution index (all indicators averaged together) when incorporated into a separate model combining age and social deprivation was significantly positively associated with both heart failure morbidity [1.39 (0.87, 1.81)] and mortality [1.79 (0.85, 2.55)] across the county.

In **Figure 2** and **Figure 4**, the left-hand maps show the unadjusted estimates of posterior total residual ward means of heart failure morbidity and mortality respectively. In **Figure 3** and **Figure 5**, the left-hand maps show the adjusted PM after multiple adjustment for the geographical location, taking into account the auto-correlation structure in the data, the uncertainty in the ward level and all ward-level risk factors (air pollution components, age, social deprivation) for heart failure morbidity and mortality respectively. The red colour indicates the maximum posterior mean recorded while green denotes the lowest mean. The right-hand maps in **Figures 2**, **3, 4** and **5** show the 95% posterior probability of heart failure and mortality, which indicate the statistical significance associated with the total excess risk. Black colour indicates a negative spatial effect (associated with increased risk of heart failure and mortality), white colour a positive effect (a decreased risk) and grey colour a non-significant effect.

In general, there was consistently higher heart failure morbidity risk in northern wards particularly around Nuneaton and Bedworth and lower heart failure morbidity risks in the

For peer review only - http://bmjopen.bmj.com/site/about/guidelines.xhtml

southern wards particularly within the district of Stratford-on-Avon. However, all this variation could partially be accounted for within the model generated within this study (taking into account air pollution, age, and social deprivation levels).

Heart failure mortality risk was, in general, higher in northern wards around Nuneaton and Bedworth as well as in some central areas around Warwick, Royal Leamington Spa, and Kenilworth. Heart failure mortality risk was again lower in more southern wards particularly within Stratford-on-Avon. Unlike with morbidity however, our model could not explain all this variation in heart failure mortality. Even after taking into account air pollution, age, and social deprivation the rate of death from heart failure remained significantly higher than would be expected in areas within and around Nuneaton, Bedworth, Warwick, Royal Leamington Spa, and Kenilworth.

In sensitivity analyses, we tested several models and our results were not substantially altered after removing one or two pollutants (data not shown).

Discussion

Before even considering air pollution, this study helps to demonstrate the inequality of risk from heart failure disease and death that exists for individuals living in different parts of the county of Warwickshire. There is a significant excess risk of both disease and death in more northern wards within and surrounding Nuneaton and Bedworth. Much (but not all) of this variation could be attributed to the air pollution, age structure and social deprivation that exists in these areas according to our model. Measures that seek to address air pollution and social deprivation could be expected therefore to help mitigate against cardiovascular risks within these local populations.

For peer review only - http://bmjopen.bmj.com/site/about/guidelines.xhtml

The present study corroborates the notion that air pollution is an increasingly important public health issue in Warwickshire. Higher levels of the average air pollution index looked at in this study correlated significantly with increased levels of both heart failure morbidity and mortality across the county, even after removing the effects of age structure and social deprivation. The individual component of NOx air pollution seems particularly to contribute risk to both the morbidity and mortality of heart failure within the county, suggesting that it may have a particularly detrimental effect on heart failure patients. This reinforces the importance of the AQMAs set up within the county in response to high NOx levels. Road traffic is a large contributor to air pollution within Warwickshire. Diesel engines are responsible for a large part of the NOx component of these emissions.

An unexpected result also appeared within our analysis. Particulate matter air pollution became significantly, negatively correlated with both heart failure morbidity and mortality when incorporated into our model with all risk factors taken into account and controlled for in this study. This would imply some sort of unexpected "protective influence" from particulate matter air pollution on heart failure patients. This clearly contradicts our expectations and is at odds with a wealth of existing evidence that indicates that particulate matter air pollution contributes risk to and exacerbates cardiovascular disease[(4)].

We offer a possible explanation for this based on the following four observations:

1. The above-mentioned negative correlation of particulate matter air pollution with heart failure morbidity and mortality in our model.
2. Particulate matter air pollution actually varied very little across the county compared to the other types of air pollution. All types of air pollution tended to decrease in rural areas, but particulate matter tended to decrease much *less* compared to the other components of air pollution. Consequently in rural areas of the county where most

For peer review only - http://bmjopen.bmj.com/site/about/guidelines.xhtml

types of air pollution are significantly lower, particulate matter pollution was relatively higher compared to NOx, Benzene, and SO_2.

3. There seems to be a high risk of heart failure deaths in urban centres (particularly Nuneaton, Bedworth, Warwick, Royal Leamington Spa, and Kenilworth), higher than can be explained by our model.
4. Conversely, there seems to be a particularly low risk of heart failure deaths in some rural areas within the western part of Stratford-on-Avon, lower than can be explained by our model.

A possible hypothesis based on these observations is that there is an additional factor influencing the morbidity and mortality of heart failure not looked at in this study, namely the urban/rural nature of a patient's living environment. It could be the case that living in an urban environment contributed risk and living in a rural area provided protection against heart failure morbidity and mortality. This would be an effect in *addition* to any increase in air pollution or social deprivation within urban settings compared to rural settings. This could certainly in principle be plausible, with people in rural areas perhaps doing more physical activity, eating more healthily etc. If this were the case it would explain the excess deaths in urban centres found in this study. It could also be responsible for the unexpected protective factor attributed to particulate matter air pollution in our analysis. Given that the particulate matter component of air pollution is *relatively* higher than the other components in rural areas, the protection that living in rural areas affords individuals could be misleadingly attributed (in our statistical analysis) to the particulate matter component of air pollution present in these areas. Further work will need to be done looking into this possible link between urban/rural living environments and heart failure morbidity and mortality. It could be very revealing to carefully characterise this effect if it indeed exists, as it may be an indication of unrecognised cardiovascular risk/protective factors associated with urban/rural living that exists within Warwickshire.

For peer review only - http://bmjopen.bmj.com/site/about/guidelines.xhtml

However, it is also important to bear in mind that this is an ecological study and all the relationships picked up between variables in this study have been found using aggregate data at the ward level (number over 50 years of age, average IMD score, average air pollution across ward, overall numbers of deaths and hospital admissions due to heart failure). It is not always a trivial task to extrapolate the conclusions drawn from such a study down to the level of individuals. Such a task would involve drilling down to individual level data and repeating the analysis, a task that was beyond the scope of this particular study. It is possible that the unexpected negative correlation between particulate matter air pollution and heart failure could disappear when data is analysed at the individual level – an example of an ecological fallacy. Consequently it would be prudent to regard the results from the individual components of air pollution with cautious interest, rather than viewing them as proof of any real effects.

However, despite these caveats, this study has been able to provide some helpful information at the population level worthy of consideration. A health inequality has been revealed, and the manner with which this inequality is influenced by age, social deprivation, and the combined index of air pollution has been demonstrated. Such information should help inform policy decisions that would influence society at a population level and hopefully improve public health in the long run.

There are some limitations in this study worth considering that result from assumptions made along the way. A single air pollution measurement in 2010 was used and it was assumed that there was no significant change in this value over the 2005-2013 periods that mortality and hospital admission data was gathered from. The resulting cross-sectional nature of the study does not allow establishing temporality and thus causality of the observed associations. There was also no way to determine the length of time that individual members of the population within a ward had lived in that area, and thus how long they had been exposed to the

For peer review only - http://bmjopen.bmj.com/site/about/guidelines.xhtml

measured ambient air pollution level. It was assumed that people with home addresses in a ward were exposed significantly to the levels of air pollution in that ward. Finally, as already mentioned, this was an ecological study, using aggregate data of risk factors to look for associations with aggregate data of morbidity and mortality. It is not always possible to apply such associations from a population level down to the individuals within that population.

In summary, this study has provided a number of interesting results. Firstly it has helped to quantify and map the inequality that exists across different parts of Warwickshire with regards to heart failure risk. It has also provided some interesting circumstantial evidence of a link between heart failure morbidity and air pollution. Finally it has also given a suggestion of a possible link between living in urban environments and a higher risk of cardiovascular disease, and a corresponding lower risk from living in rural environments. More work will need to be done to look into this particular possibility. It would be informative to run this type of analysis whilst factoring in the influence of a person's distance from their nearest urban centre. This urban/rural factor should be further explored and mined for additional information as it could be an indication of hitherto unconsidered factors influencing the health status of the population of Warwickshire and possibly further afield.

In order to determine the validity of our conclusions at the individual level further work would need to be done analysing the available data from individual patients (risks and outcomes). Such work could help to characterise the true effect of different components of air pollution at the individual level. It would also be interesting to determine if the different components of air pollution act as effect modifiers on each other.

It would be possible to look at the effects of air pollution variation in the shorter term as well. For example, looking at how local "spikes" in air pollution affect the rates of hospital admissions locally immediately following it. This could be done in Leamington Spa where there is an air quality monitoring station constantly measuring the levels of air pollutants.

For peer review only - http://bmjopen.bmj.com/site/about/guidelines.xhtml

Other health problems, such as ischaemic heart disease and respiratory diseases, have been linked with air pollution as well and it could be informative to also look into these links locally.

Contributorship statement:

OB conceived the idea, analyzed the data, contributed to formulating the results and wrote the first draft. N-B K analyzed the data, advised on statistical aspects, contributed to formulating the results, wrote the second draft. CJ analyzed the data. JL helped coordinate the project and co-wrote the final draft; AC coordinated the project, advised on all aspects and co-wrote the final draft.

Funding:

This paper presents independent research supported by the National Institute for Health Research (NIHR) Collaborations for Leadership in Applied Health Research and Care West Midlands. The views expressed are those of the author(s) and not necessarily those of the NHS, the NIHR or the Department of Health.

Competing interests: The authors declared no conflict of interest.

Data sharing: Not application

References

1. Particulate Air Pollution and Hospital Emergency Room Visits for Asthma in Seattle, Joel Schwartz, Daniel Slater, Timothy V. Larson, William E. Pierson, and Jane Q. Koenig. American Review of Respiratory Disease, Vol. 147, No. 4 (1993), pp. 826-831.
2. Global association of air pollution and heart failure: a systematic review and meta-analysis, Anoop SV Shah, Jeremy P Langrish,Harish Nair,David A McAllister, Amanda L Hunter, Prof Ken Donaldson, Prof David E Newby, Dr Nicholas L Mills. The Lancet, Early Online Publication, 10 July 2013 DOI: 10.1016/S0140-6736(13)60898-3
3. Air pollution and lung cancer incidence in 17 European cohorts: prospective analyses from the European Study of Cohorts for Air Pollution Effects (ESCAPE), Raaschou-Nielsen O, Andersen ZJ, et al. Lancet Oncol. 2013 Aug;14(9):813-22
4. Cardiovascular Mortality and Long-Term Exposure to Particulate Air Pollution, Epidemiological Evidence of General Pathophysiological Pathways of Disease C. Arden Pope III, Richard T. Burnett, et al. Circulation. 2004; 109: 71-77

For peer review only - http://bmjopen.bmj.com/site/about/guidelines.xhtml

5. Air pollution and health, Bert Brunekreef, Stephen T Holgate, The Lancet, Volume 360, Issue 9341, 19 October 2002, Pages 1233–1242
6. Department of Environment, Food and Rural Affairs (DEFRA), department website, weblink (accessed 2/12/13): http://aqma.defra.gov.uk/aqma/local_authorities.php?la_id=296
7. Epidemiology, pathophysiology, and etiology of congestive heart failure in older adults. Rich MW. J Am Geriatr Soc. 1997 Aug;45(8):968-74.
8. Influence of socioeconomic deprivation on the primary care burden and treatment of patients with a diagnosis of heart failure in general practice in Scotland: population based study. F A McAlister, N F Murphy, et al. BMJ. 2004 May 8; 328(7448): 1110.
9. Warwickshire Observatory website – ONS population estimates. Web link (accessed on 3/10/13): http://www.warwickshireobservatory.org/observatory/observatorywcc.nsf/05a17cfbd3bcf85d802572920033cf0f/bfb8b0a1d34f274c802572c00048108f?OpenDocument
10. Deprivation data produced by the Department for Communities and Local Government. File located at web link (accessed on 3/10/13): https://www.gov.uk/government/publications/english-indices-of-deprivation-2010 Ward based population weighted averages calculated from LSOA specific data by the London Health Observatory and North East Public Health Observatory.
11. Fahrmeir L, Lang S. Bayesian Inference for Generalized Additive Mixed Models Based on Markov Random Field Priors. *Applied Statistics (JRSS C)*. 2001;50:201-20.
12. Kandala N-B, Tigbe W, Manda S, Stranges S. Spatial Variation of Predictors of Hypertension in Sub-Saharan Africa: A Case Study of South-Africa. American Journal of Hypertension 2013. 10.1093/ajh/hps063.
13. Belitz C, Brezger A, Kneib T, Lang S. BayesX Software for Bayesian Inference in Structured Additive Regression Models Version 2.0.1, 2012. Accessed at: http://www.stat.uni-muenchen.de/~bayesx/manual/methodology_manual.pdf
14. Taken from the Warwickshire Observatory Quality of Life in Warwickshire 2012/13 report. Weblink (accessed on 3/10/13): http://maps.warwickshire.gov.uk/iareports/environmental_issues/

Author's contributions

OB conceived the idea, analyzed the data, contributed to formulating the results and wrote the first draft.

N-B K analyzed the data, advised on statistical aspects, contributed to formulating the results, wrote the second draft. CJ analyzed the data. JL helped coordinate the project and co-wrote the final draft; AC coordinated the project, advised on all aspects and co-wrote the final draft.

For peer review only

For peer review only - http://bmjopen.bmj.com/site/about/guidelines.xhtml

Figure legends

Figure 1 : Warwickshire map with 2010 air quality index (all components of air pollution combined) displayed by ward (left; Warwickshire map with number of heart failure hospital admissions per 1000 population between 2005 – 2013 displayed by ward(centre) and Warwickshire map with total heart failure deaths per 1000 population between 2007 – 2012 displayed by ward(right).

[FIGURE 1 ABOUT HERE]

Figure 2: Left: Unadjusted total residual spatial effects of morbidity risk associated with heart failure, at ward level in Warwickshire. Shown are the posterior means. Right: Corresponding posterior probabilities at 80% nominal level.

[FIGURE 2 ABOUT HERE]

Red coloured – high risk
Green coloured – low risk

Black coloured – significant positive spatial effect
White coloured- significant negative spatial effect
Grey coloured – no significant effect

Figure 3: Left: Total residual spatial effects of morbidity risk associated with heart failure, at ward level in Warwickshire. Shown are the posterior means of the full model (IMD 2010, Over 50, and the 4 indicators of air pollution). Right: Corresponding posterior probabilities at 80% nominal level.

[FIGURE 3 ABOUT HERE]

Red coloured – high risk
Green coloured – low risk

Black coloured – significant positive spatial effect
White coloured- significant negative spatial effect
Grey coloured – no significant effect

Figure 4: Left: Unadjusted total residual spatial effects of mortality risk associated with heart failure, at ward level in Warwickshire. Shown are the posterior means. Right: Corresponding posterior probabilities at 80% nominal level.

[FIGURE 4 ABOUT HERE]

Red coloured – high risk
Green coloured – low risk

Black coloured – significant positive spatial effect
White coloured- significant negative spatial effect
Grey coloured – no significant effect

Figure 5: Left: Total residual spatial effects of mortality risk associated with heart failure, at ward level in Warwickshire. Shown are the posterior means of the full model (IMD 2010, Over 50, and the 4 indicators of air pollution). Right: Corresponding posterior probabilities at 80% nominal level.

[FIGURE 5 ABOUT HERE]

Red coloured – high risk
Green coloured – low risk

Black coloured – significant positive spatial effect
White coloured- significant negative spatial effect
Grey coloured – no significant effect

Figure 6 – Warwickshire map displaying the 2010 levels of deprivation (expressed as Multiple Deprivation Scores) by LSOAs. Produced by the Warwickshire Observatory (left) and Warwickshire map displaying the 2008 midyear estimates of % of people over the age of 50 by SOA. Produced by the Warwickshire Observatory (right).

For peer review only - http://bmjopen.bmj.com/site/about/guidelines.xhtml

For peer review only

Table 1: Posterior mean (PM) of fixed effects estimates of heart failure admission and mortality across air pollutions indicators (Warwickshire 2005-2013)

**Variable**	Heart failure admission PM & 95%CI‡	Heart failure mortality PM & 95%CI †
Constant	6.19 (3.84, 8.97)	4.13 (1.18, 7.43)
Nitrogen dioxide indicator (No2)	3.35 (1.89, 4.99)	4.30 (1.68, 7.37)
Sulphur dioxide indicator (So2))	7.75 (-3.84, 17.99)	11.02 (-1.21, 22.41)
Particulates indicator (Pm)	-12.93 (-20.41, -6.54)	-14.69 (-23.46, -6.50)
Benzene indicator (Ben)	[31.9:8.36, 55.85]	25.98 (-6.62, 54.22)
Over 50 years %	0.70(-0.63, 1.98)	1.60, (0.47, 2.92)
IMD 2010 score	0.02 (0.01, 0.03)	0.00 (-0.01, 0.01)

‡ Spatially adjusted posterior mean (PM) from Bayesian geo-additive regression models after controlling for fixed effect of all air pollutions indicators: Mono-nitrogen Oxide (NOx), sulphur dioxide (So2), particulate matter (Pm), benzene (Ben) and combined index and the county of residence (spatial effects). †Spatially adjusted posterior mean (PM) from Bayesian geo-additive regression models after controlling for fixed effect of all air pollutions indicators: Mono-nitrogen Oxide (NOx), sulphur dioxide (So2), particulate matter (Pm), benzene (Ben) and combined index and the county of residence (spatial effects).

For peer review only

For peer review only - http://bmjopen.bmj.com/site/about/guidelines.xhtml

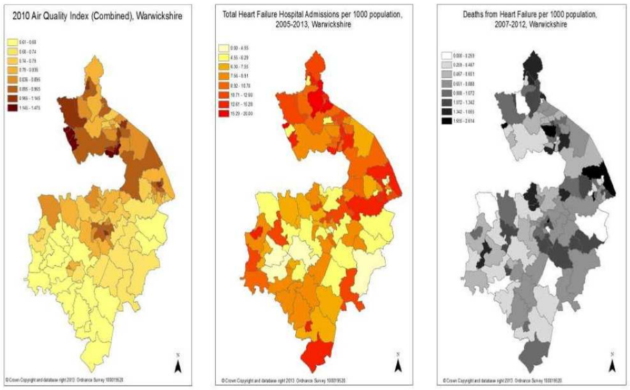

209x297mm (300 x 300 DPI)

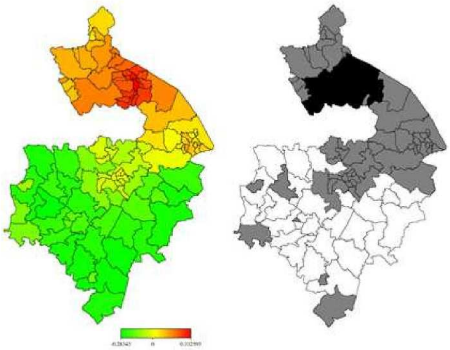

209x297mm (300 x 300 DPI)

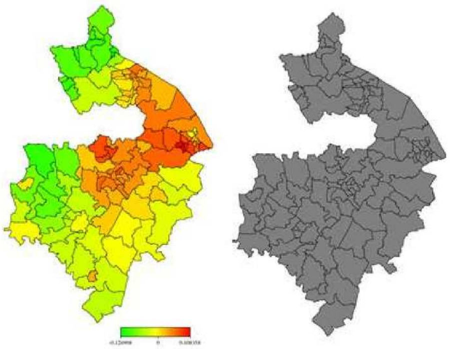

209x297mm (300 x 300 DPI)

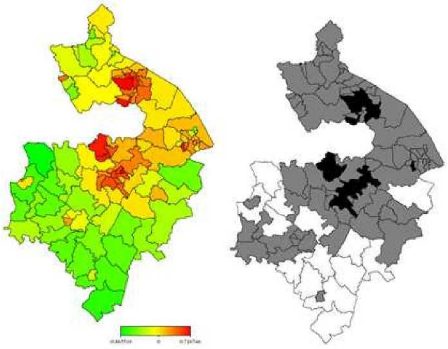

209x297mm (300 x 300 DPI)

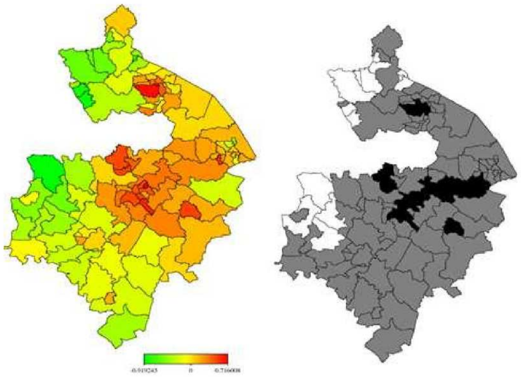

209x297mm (300 x 300 DPI)

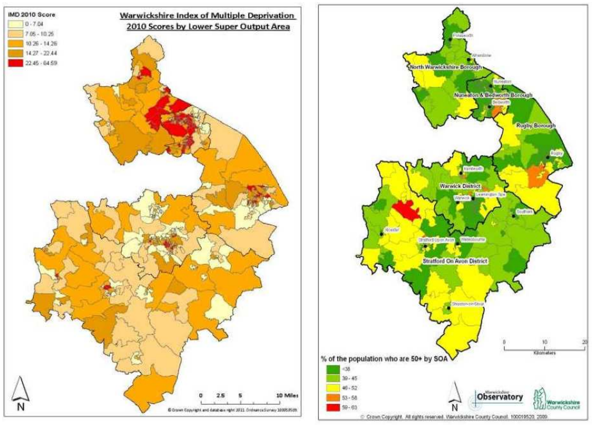

209x297mm (300 x 300 DPI)

STROBE 2007 (v4) Statement—Checklist of items that should be included in reports of *cross-sectional studies*

Section/Topic	Item #	Recommendation	Reported on page #
**Title and abstract**	1	(*a*) Indicate the study's design with a commonly used term in the title or the abstract	2
		(*b*) Provide in the abstract an informative and balanced summary of what was done and what was found	2
**Introduction**			
Background/rationale	2	Explain the scientific background and rationale for the investigation being reported	4
Objectives	3	State specific objectives, including any prespecified hypotheses	4,5
**Methods**			
Study design	4	Present key elements of study design early in the paper	4, 5
Setting	5	Describe the setting, locations, and relevant dates, including periods of recruitment, exposure, follow-up, and data collection	5,6
Participants	6	(*a*) Give the eligibility criteria, and the sources and methods of selection of participants	5,6,
Variables	7	Clearly define all outcomes, exposures, predictors, potential confounders, and effect modifiers. Give diagnostic criteria, if applicable	5-7
Data sources/ measurement	8*	For each variable of interest, give sources of data and details of methods of assessment (measurement). Describe comparability of assessment methods if there is more than one group	5-6
Bias	9	Describe any efforts to address potential sources of bias	*7-8,12*
Study size	10	Explain how the study size was arrived at	5
Quantitative variables	11	Explain how quantitative variables were handled in the analyses. If applicable, describe which groupings were chosen and why	7-8
Statistical methods	12	(*a*) Describe all statistical methods, including those used to control for confounding	7,8
		(*b*) Describe any methods used to examine subgroups and interactions	NA
		(*c*) Explain how missing data were addressed	NA
		(*d*) If applicable, describe analytical methods taking account of sampling strategy	NA
		(*e*) Describe any sensitivity analyses	NA
**Results**			

For peer review only

Participants	13*	(a) Report numbers of individuals at each stage of study—eg numbers potentially eligible, examined for eligibility, confirmed eligible, included in the study, completing follow-up, and analysed	2, 5,6
		(b) Give reasons for non-participation at each stage	NA
		(c) Consider use of a flow diagram	NA
Descriptive data	14*	(a) Give characteristics of study participants (eg demographic, clinical, social) and information on exposures and potential confounders	6
		(b) Indicate number of participants with missing data for each variable of interest	NA
Outcome data	15*	Report numbers of outcome events or summary measures	10-11
Main results	16	(*a*) Give unadjusted estimates and, if applicable, confounder-adjusted estimates and their precision (eg, 95% confidence interval). Make clear which confounders were adjusted for and why they were included	8
		(*b*) Report category boundaries when continuous variables were categorized	NA
		(*c*) If relevant, consider translating estimates of relative risk into absolute risk for a meaningful time period	NA
Other analyses	17	Report other analyses done—eg analyses of subgroups and interactions, and sensitivity analyses	NA
**Discussion**			
Key results	18	Summarise key results with reference to study objectives	8-10
Limitations	19	Discuss limitations of the study, taking into account sources of potential bias or imprecision. Discuss both direction and magnitude of any potential bias	11, 15
Interpretation	20	Give a cautious overall interpretation of results considering objectives, limitations, multiplicity of analyses, results from similar studies, and other relevant evidence	11-15
Generalisability	21	Discuss the generalisability (external validity) of the study results	11-15
**Other information**			
Funding	22	Give the source of funding and the role of the funders for the present study and, if applicable, for the original study on which the present article is based	NA

*Give information separately for cases and controls in case-control studies and, if applicable, for exposed and unexposed groups in cohort and cross-sectional studies.

Note: An Explanation and Elaboration article discusses each checklist item and gives methodological background and published examples of transparent reporting. The STROBE checklist is best used in conjunction with this article (freely available on the Web sites of PLoS Medicine at http://www.plosmedicine.org/, Annals of Internal Medicine at http://www.annals.org/, and Epidemiology at http://www.epidem.com/). Information on the STROBE Initiative is available at www.strobe-statement.org.

For peer review only
